# Resting vs. active: a meta‐analysis of the intra‐ and inter‐specific associations between minimum, sustained, and maximum metabolic rates in vertebrates

**DOI:** 10.1111/1365-2435.12879

**Published:** 2017-05-02

**Authors:** Sonya K. Auer, Shaun S. Killen, Enrico L. Rezende

**Affiliations:** ^1^ Institute of Biodiversity, Animal Health and Comparative Medicine University of Glasgow Graham Kerr Building Glasgow G12 8QQ UK; ^2^ Facultad de Ecología y Recursos Naturales Universidad Andres Bello Santiago Chile

**Keywords:** aerobic capacity, daily energy expenditure, locomotion, maximum thermogenesis, resting metabolic rate, standard metabolic rate

## Abstract

1. Variation in aerobic capacity has far reaching consequences for the physiology, ecology, and evolution of vertebrates. Whether at rest or active, animals are constrained to operate within the energetic bounds determined by their minimum (minMR) and sustained or maximum metabolic rates (upperMR). MinMR and upperMR can differ considerably among individuals and species but are often presumed to be mechanistically linked to one another. Specifically, minMR is thought to reflect the idling cost of the machinery needed to support upperMR. However, previous analyses based on limited datasets have come to conflicting conclusions regarding the generality and strength of their association.
2. Here we conduct the first comprehensive assessment of their relationship, based on a large number of published estimates of both the intra‐specific (*n* = 176) and inter‐specific (*n* = 41) phenotypic correlations between minMR and upperMR, estimated as either exercise‐induced maximum metabolic rate (VO_2_max), cold‐induced summit metabolic rate (Msum), or daily energy expenditure (DEE).
3. Our meta‐analysis shows that there is a general positive association between minMR and upperMR that is shared among vertebrate taxonomic classes. However, there was stronger evidence for intra‐specific correlations between minMR and Msum and between minMR and DEE than there was for a correlation between minMR and VO_2_max across different taxa. As expected, inter‐specific correlation estimates were consistently higher than intra‐specific estimates across all traits and vertebrate classes.
4. An interesting exception to this general trend was observed in mammals, which contrast with birds and exhibit no correlation between minMR and Msum. We speculate that this is due to the evolution and recruitment of brown fat as a thermogenic tissue, which illustrates how some species and lineages might circumvent this seemingly general association.
5. We conclude that, in spite of some variability across taxa and traits, the contention that minMR and upperMR are positively correlated generally holds true both within and across vertebrate species. Ecological and comparative studies should therefore take into consideration the possibility that variation in any one of these traits might partly reflect correlated responses to selection on other metabolic parameters.

A lay summary is available for this article.

## Introduction

Metabolism is the ‘fire of life’ that fuels processes at all levels of biological organization (Kleiber 1961) and has far reaching consequences for the physiology, behaviour, ecology, and evolution of organisms (Chown & Gaston 1999; Brown *et al*. 2004; Anderson & Jetz 2005; Buckley, Rodda & Jetz 2008). Metabolic rates vary widely across individuals, populations, and species (Burton *et al*. 2011; White & Kearney 2013; Killen *et al*. 2016). They are heritable to a certain extent (Nespolo *et al*. 2005; Nilsson, Åkesson & Nilsson 2009; Wone *et al*. 2009) and can evolve in both laboratory (Książek, Konarzewski & Łapo 2004; Wone *et al*. 2015) and wild populations (Boratyński & Koteja 2010). Metabolic rates are often under selection (Hayes & O'Connor 1999; Bochdansky *et al*. 2005) and their variation among individuals has been linked to components of fitness such as growth (Steyermark 2002; Auer *et al*. 2015b), reproduction (Blackmer *et al*. 2005; Boratyński & Koteja 2010) and survival (Artacho & Nespolo 2009; Larivee *et al*. 2010). Indeed, inter‐specific variation in metabolic rates has been attributed to a wide range of extrinsic factors such as climate, lifestyle, habitat productivity, and diet (Mueller & Diamond 2001; Rezende *et al*. 2004; Anderson & Jetz 2005; Bozinovic *et al*. 2009; White & Kearney 2013; Killen *et al*. 2016).

The links between the lower and upper limits to energy expenditure (minMR and upperMR hereafter) have garnered significant interest over the last half century. The baseline energetic costs of living are set by minMR (Hulbert & Else 2004), which have been quantified as standard metabolic rate (SMR) in ectotherms, basal metabolic rate (BMR) in endotherms, or simply resting metabolic rate (RMR) as a proxy for the previous estimates under less restrictive conditions (e.g., allowing for low levels of spontaneous activity and some digestion; Jobling 1994). In contrast, upperMR sets the limit for the energy available to finance locomotion, digestion, growth, reproduction, and thermoregulation. UpperMR has been quantified acutely as maximum metabolism during strenuous exercise (VO_2_max) or cold‐exposure for endothermic organisms (summit metabolism or Msum) and, over longer time spans, as sustained metabolic rates and daily energy expenditure (DEE). Early observations that minMR appears to be a relatively constant proportion of both sustained and maximum metabolic rates (Bennett & Ruben 1979; Drent & Daan 1980; Hammond & Diamond 1997) led to the hypothesis that they are mechanistically linked (Packard 1968; Bennett & Ruben 1979; Drent & Daan 1980; Taigen 1983; Hayes & Garland 1995) and may evolve together in a correlated fashion (Hayes 2010; Nespolo *et al*. 2017). This premise underlies various models such as the ‘aerobic capacity model for the evolution of endothermy’ (Bennett & Ruben 1979), the ‘assimilation capacity model for the evolution of endothermy’ (Koteja 2000), and the ‘sustained maximal limit model’ (Drent & Daan 1980; Speakman, Król & Johnson 2004), which posit that minMR reflects the idling cost of maintaining the machinery required to support total energy expenditure.

An association between minMR and upperMR across vertebrate lineages has important implications for their ecological and evolutionary physiology. Not only does it open up the question of which cellular or tissue‐level mechanisms determine or limit different aspects of aerobic performance (Chappell *et al*. 2007; Norin & Malte 2012), but also how organisms might respond to different and often antagonistic selective pressures (e.g. Rezende *et al*. 2004; Killen *et al*. 2016). Not surprisingly, many studies have estimated the correlation between these traits at both the intra‐ and inter‐specific level, but with mixed results. For instance, studies have reported positive and nonsignificant phenotypic correlations at the inter‐specific level (e.g. Ricklefs, Konarzewski & Daan 1996; Rezende *et al*. 2004) and positive, negative, and nonsignificant correlations at the intra‐specific level (e.g. Gomes *et al*. 2004; Rezende *et al*. 2005; Dlugosz *et al*. 2012). Genetic correlations between minMR and upperMR (Dohm, Hayes & Garland 2001; Sadowska *et al*. 2005; Wone *et al*. 2009) and correlated responses to selection (Książek, Konarzewski & Łapo 2004; Sadowska *et al*. 2015) also provide equivocal results. No consensus seems to emerge because studies focus on different taxonomic groups (from fish to birds and mammals), metabolic traits (VO_2_max, Msum, and DEE) and levels of organization (intra‐ vs. inter‐specific). Hence, it is currently unclear whether there are systematic factors that explain variation in the degree to which minMR and upperMR are linked.

**Figure 1:**
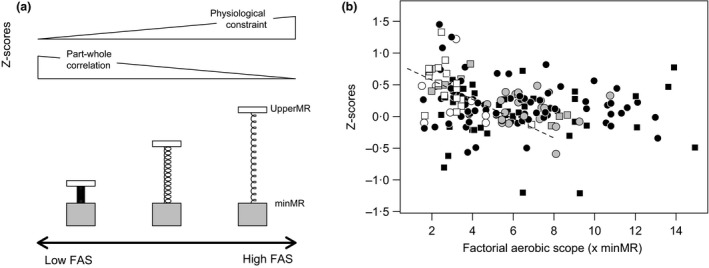
(a) Conceptual model: The strength of association between minMR and upperMR should vary predictably with factorial aerobic scope (FAS = upperMR/minMR), as shown with the coil spring model, either because species with high FAS are near a physiological limit (stretched spring) or due to part‐whole correlation because minMR encompasses an increasingly large fraction of upperMR in species with lower FAS (compressed spring). (b) Empirical evaluation: *Z*‐scores of the correlation between minMR and each of exercise‐induced maximum metabolic rate (black), cold‐induced summit metabolic rate (grey) and daily energy expenditure (white). Data are from this study. Correlations were assessed using different measures of minMR: standard and basal metabolic rate (circles) or resting metabolic rate (squares). Our analyses support the later alternative for correlations involving DEE, as shown by the dotted line.

Whether the association between minMR and upperMR is driven by physiological constraint or statistical artefact is also not well understood. Selection for high aerobic performance is thought to result in increased metabolic expenditure at rest, which inherently assumes that a positive correlation emerges from a physiological constraint (i.e., upperMR drives the correlation; Bennett & Ruben 1979; Drent & Daan 1980; Taigen 1983; Hayes & Garland 1995). However, part‐whole correlation between minMR and upperMR may also give rise to a positive correlation (for a simple algebraic formulation, see Chayes 1971); if energy requirements for different processes such as locomotion are additive to minMR (i.e. minMR is not suppressed during periods of high energy expenditure), then upperMR could simply reflect minMR rather than aerobic performance that is independent of minMR (Ricklefs, Konarzewski & Daan 1996; Speakman 2000; Pontzer, Brown & Raichlen 2016). These alternatives can be disentangled by assessing whether correlations exhibit a positive vs. negative association with FAS (Fig. 1a). If species with a high FAS are closer to a physiological limit in which upperMR may not increase any further without also increasing minMR, as proposed by the aerobic capacity model (Bennett & Ruben 1979), then correlations are expected to be positively associated with FAS (stretched coil spring in Fig. 1a). Alternatively, if the relationship between minMR and upperMR is due to part‐whole correlation, then correlations are expected to be negatively associated with FAS because minMR becomes increasingly correlated with itself as FAS decreases (compressed coil spring in Fig. 1a).

Here we address these issues by using a meta‐analytical approach to examine the large number of published estimates of both the intra‐specific and inter‐specific phenotypic correlations between minMR and upperMR. Our specific objectives were to determine: (i) the magnitude and direction of intra‐ and inter‐specific correlations and whether they vary across traits since VO_2_max, Msum, and DEE represent very different measures of metabolism that are not necessarily correlated across individuals (Peterson, Walton & Bennett 1998; Chappell *et al*. 2004, 2007; Swanson *et al*. 2012) or species (Wiersma, Chappell & Williams 2007); (ii) whether correlations vary predictably among vertebrate taxonomic classes; (iii) the level of agreement between correlations reported at intra‐ and inter‐specific levels; and (iv) whether the strength of the association between minMR and upperMR is driven by physiological constraint or statistical artefact, by looking for directional trends between the magnitude of the correlation and the factorial difference between minMR and upperMR.

## Materials and methods

### Literature review and selection criteria

We searched for published estimates of intra‐specific and inter‐specific phenotypic correlations between the different types of minMR and upperMR using both Google Scholar and Web of Science. We used the following search terms: intra‐specific, inter‐specific, correlation, metabolic rate, metabolism, energy expenditure, standard metabolism, standard metabolic rate, basal metabolism, basal metabolic rate, resting metabolism, resting metabolic rate, daily energy expenditure, field metabolism, field metabolic rate, maximum metabolism, maximum metabolic rate, summit metabolism, summit metabolic rate, peak metabolism, and the related acronyms RMR, SMR, BMR, DEE, FMR, MMR, VO_2_max and Msum. We also searched the reference list of each paper to identify additional studies missed in our initial search. Finally, several unpublished estimates of intra‐specific correlations were obtained from colleagues at the University of Glasgow. Correlations among measures of metabolic rate may be positive simply because they are all highly dependent on body mass. Thus, only phenotypic correlations that accounted for variation in body mass were considered.

For each study, we recorded the species name (for intra‐specific correlations) and taxonomic class (fish, amphibian, reptile, bird or mammal). We also recorded the sample size of the study, the type of minMR (RMR vs. SMR or BMR) and upperMR (VO_2_max, Msum and DEE) measured, the estimate of their correlation (Pearson's *r*) and the factorial aerobic scope associated with each correlation as an estimate of the factorial difference between minMR and upperMR (FAS = upperMR/minMR). The respirometry methods for many studies were not detailed enough for us to assess whether RMR vs. SMR or BMR was being measured, so labels provided in the original studies were used. DEE was typically measured using the doubly labelled water technique, but several studies measured the average daily oxygen consumption of animals living in the laboratory (e.g. Chappell *et al*. 2004). For studies that did not provide the correlation estimate, we obtained it from reported *P*‐values and *t* or *F* statistics, by contacting the authors directly, or by using data grabbing software (Graphclick; http://www.arizona-software.ch/graphclick/). For inter‐specific studies, we recorded whether analyses accounted for phylogenetic history.

### Statistical analyses

Meta‐analyses of intra‐specific and inter‐specific correlations were performed with the statistical package metafor for r (Viechtbauer 2010), employing Fisher's *r*‐to‐*z* transformation to obtain unbiased estimates of effect sizes and sampling variances (Hedges & Olkin 1985). We used Akaike's information criterion (AIC) to compare models with different fixed effects, and a multi‐step approach employing ML for model selection and REML for the estimation of variance components of the best candidate models (see Ngo & Brand 1997 and references therein). We used the AIC_c_ corrected for small sample sizes for model selection, and quantified the relative support of each model with Akaike's weights (*w*_i_). The best models were those whose Akaike weights were within 10% of the highest value in each set (see below), a minimum cut‐off point comparable to that suggested by Royall (1997). Given their different underlying physiology and ecological significance (McKechnie & Swanson 2010), VO_2_max, Msum, and DEE were analyzed separately and the 95% confidence intervals for intra‐specific and inter‐specific effect sizes were estimated from these models for each taxonomic class.

**Figure 2:**
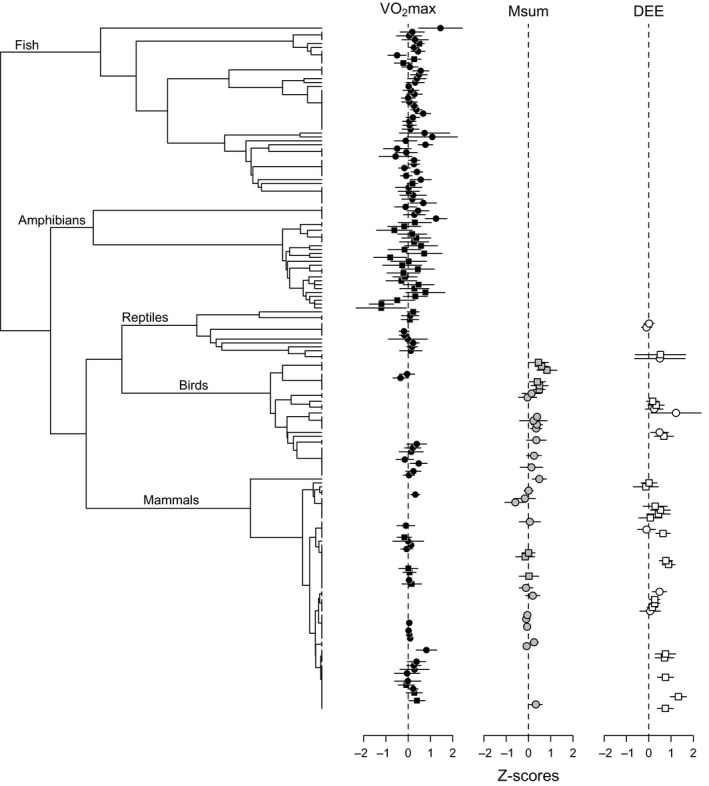
Phylogeny and distribution of effect sizes for the intra‐specific correlation between minMR and exercise‐induced maximum metabolic rate (VO_2_max), cold‐induced summit metabolic rate (Msum), and daily energy expenditure (DEE). Correlations were assessed using different measures of minMR: standard and basal metabolic rate (circles) or resting metabolic rate (squares). See Appendix S1 for details on species and their phylogenetic relationships and Appendix S2 for species’ correlations and references.

For analyses of intra‐specific correlations, we assembled a dated phylogeny based on different sources in the literature (Fig. 2, see Appendix S1, Supporting Information). The impact of phylogeny was determined by estimating the λ (Pagel 1999) that best fit the residual variation of models including species as a random factor (since multiple studies reported correlations for the same species). For each of VO_2_max, Msum, and DEE, a standard model with the type of minMR measured (SMR or BMR vs. RMR) included as a categorical factor was compared against more complex models including taxonomic class and FAS. Because minMR type (SMR or BMR vs. RMR) had a negligible impact, the mean effect of this factor was employed to calculate adjusted effect sizes and their 95% confidence intervals. Some studies provided multiple estimates, so study was initially included as a random effect in all analyses. However, it was subsequently removed since in all cases it did not improve the model fit.

For inter‐specific analyses, we examined the effects of taxonomic class and a categorical variable coding phylogenetic vs. non‐phylogenetic analyses. In this case, we did not use a dummy variable coding for RMR vs. BMR or SMR since some studies included both estimates and controlled for them statistically in their analyses (e.g. Rezende *et al*. 2004). Importantly, some studies may have included the same species data in their analyses (e.g., the data for passerines in Dutenhoffer & Swanson 1996 constitutes a subset of the dataset compiled by Rezende *et al*. 2002 for birds), and therefore some degree of pseudo‐replication is expected between results. Because the degree of overlap between datasets varies from study to study and may not be readily removed without the raw data, we opted to include all analyses compiled for completeness. Nonetheless, the adjusted estimates and confidence intervals reported here must be interpreted with caution in light of this limitation.

## Results

**Figure 3:**
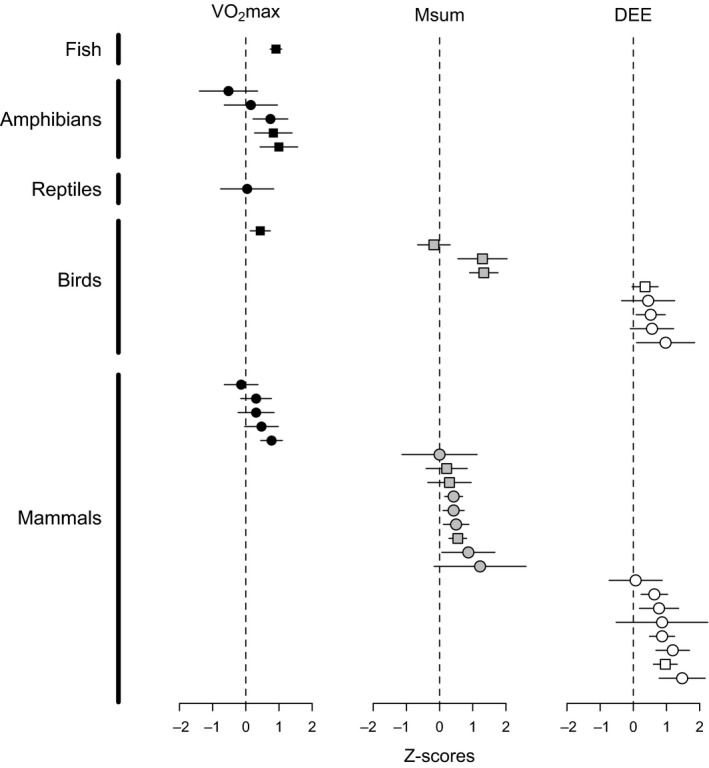
Effect sizes for the inter‐specific correlation between minimum metabolic rate and exercise‐induced maximum metabolic rate (VO_2_max), cold‐induced summit metabolic rate (Msum) or daily energy expenditure (DEE). Correlations in original studies were assessed with phylogenetic (squares) and non‐phylogenetic analyses (circles). See Appendix S3 for details of taxonomic classes and references.

We obtained a total of 176 estimates of intra‐specific phenotypic correlations and 41 estimates of inter‐specific correlations between minMR and either VO_2_max, Msum, or DEE (Table 1, Appendices S2 and S3). For the intra‐specific dataset, we obtained estimates for 73 different species from a total of 75 studies, which included 115 correlations with VO_2_max across all taxa, 31 correlations with Msum across birds and mammals, and 30 correlations with DEE across reptiles, birds, and mammals (Fig. 2, Appendix S2). In contrast, the inter‐specific dataset contained a total of 15 published papers that included 14 correlation estimates for VO_2_max, 12 for Msum, and 15 for DEE (Fig. 3, Appendix S3). Funnel plots of effect size as a function of log_10_‐transformed sample size were symmetrical for both intra‐specific and inter‐specific correlations estimates (Appendix S4), suggesting a lack of publication bias.

**Table 1:** Summary for each taxonomic class of studies examining the intra‐specific and inter‐specific correlations between minimum metabolism and exercise‐induced maximum metabolic rate (VO_2_max), cold‐induced summit metabolic rate (Msum), and daily energy expenditure (DEE)

	Studies	Species	*N*	VO_2_max	Msum	DEE
Intra‐specific						
Fish	22	19	6–452	46	0	0
Amphibians	3	20	6–19	27	0	0
Reptiles	8	8	6–250	9	0	4
Birds	15	12	6–200	9	15	6
Mammals	27	14	11–1334	24	16	20
Inter‐specific						
Fish	1	131	–	1	0	0
Amphibians	5	8–17	–	5	0	0
Reptiles	1	9	–	1	0	0
Birds	9	8–45	–	1	3	5
Mammals^a^	25	4–60	–	6	9	10

See Appendices S2 and S3 for details of species and references. Listed are the numbers of studies, species, individuals per correlation (*N*), and correlations based on each of VO_2_max, Msum and DEE.

a One correlation for DEE and one for VO_2_max were not included in inter‐specific analyses because they were calculated employing four species, hence *z*‐scores and variance estimates could not be calculated (see Table 3).

### Intra‐specific correlations

**Figure 4:**
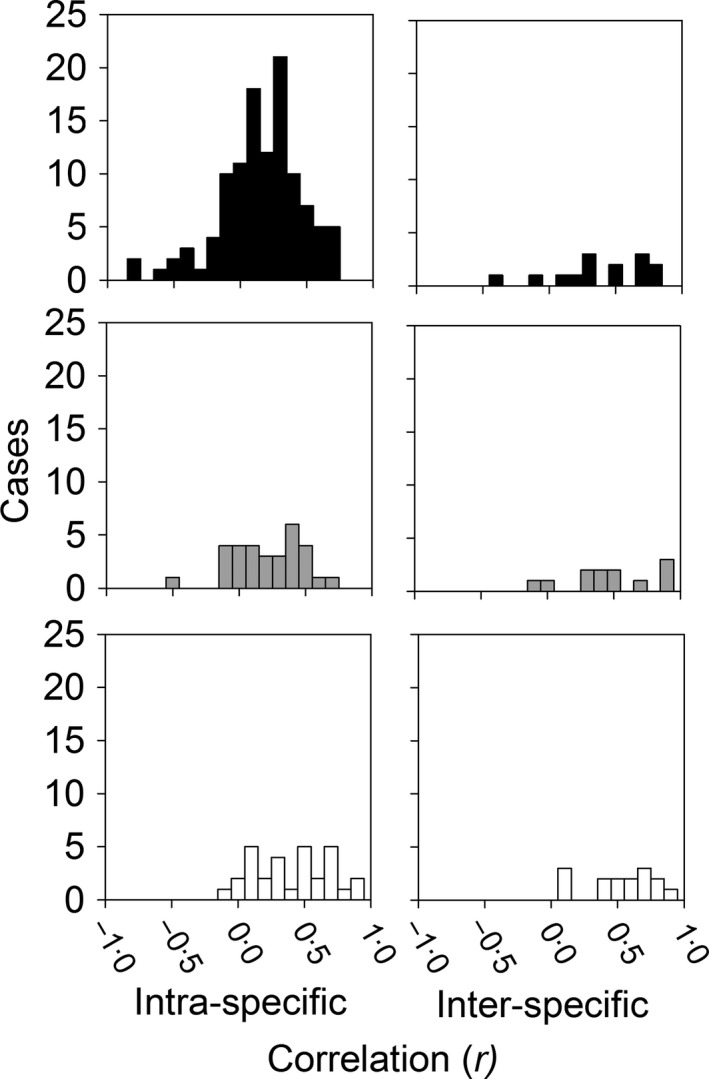
Frequency distributions of the intra‐ and inter‐specific correlations between minimum metabolic rate (standard, basal, and resting) and each of exercise‐induced maximum metabolic rate (VO_2_max = black), cold‐induced maximum metabolic rate (Msum = grey), and daily energy expenditure (DEE = white) in vertebrates.

**Figure 5:**
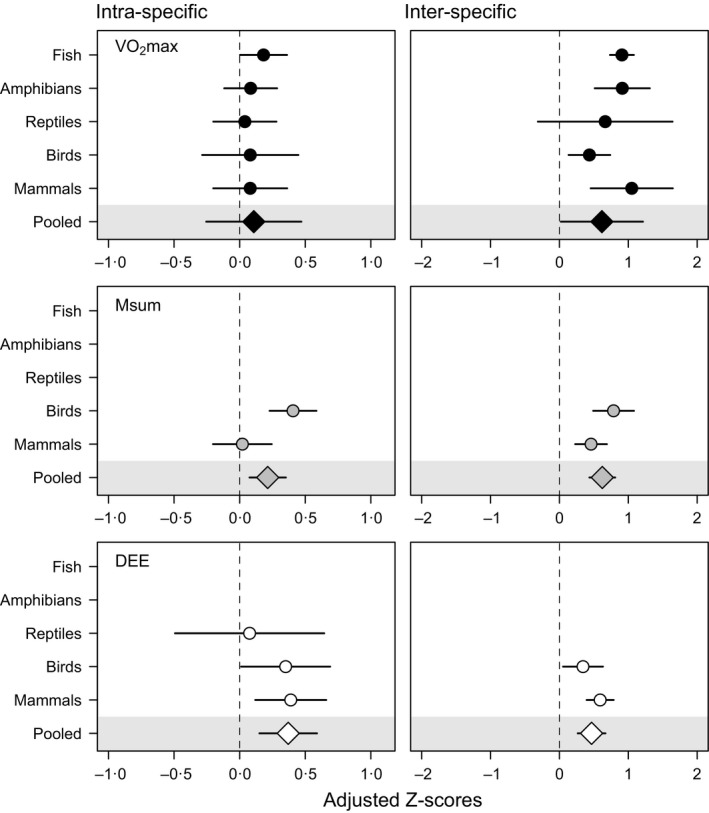
Means and 95% confidence intervals for effect sizes of the intra‐ and inter‐specific correlation between minimum metabolic rate and exercise‐induced maximum metabolic rate (VO_2_max), cold‐induced summit metabolic rate (Msum), and daily energy expenditure (DEE). Because inter‐specific estimates often differed between phylogenetic vs. non‐phylogenetic analyses (see Results), adjusted effect sizes were calculated for phylogenetic analyses.

Pearson's correlation coefficients for the intra‐specific dataset ranged from −0·837 to 0·896 for VO_2_max, from −0·530 to 0·680 for Msum, and from −0·129 to 0·869 for DEE (Figs 2 and 4). For VO_2_max, comparison between models suggests that there is a small amount of phylogenetic signal in effect sizes (λ = 0·1) that seems to partly reflect differences among taxonomic classes, since λ = 0·0 in models including class as a predictor (Table 2). Based on AIC_c_ estimates, neither the inclusion of class nor FAS improved overall fit, and the distribution of effect sizes suggests the correlation between minMR and VO_2_max is very close to zero in all taxonomic groups (Fig. 5). In contrast, effect sizes with Msum exhibited strong phylogenetic signal (λ = 0·5 in the standard model) and were consistently positive for birds but not mammals (Fig. 5). Accordingly, based on AIC_c_ estimates, the two models with the best fit include taxonomic class as a predictor (Table 2). For DEE, effect sizes did not exhibit phylogenetic signal (λ = 0·0) and were generally positive (Fig. 5). Even though no differences were evident between taxonomic classes, comparisons between models suggest that effect sizes involving DEE may be influenced by FAS (Table 2). Accordingly, there was a significant negative relationship between effect sizes and FAS (Fig. 1b; *z *=* *−2·82, *P *=* *0·005) in the model including this predictor. Taken together, our analyses suggest that intra‐specific correlations are generally positive for Msum and DEE but not VO_2_max (Fig. 5). In addition, the magnitude of correlations between minMR and DEE decreases with FAS whereas no association is evident for VO_2_max or Msum (Fig. 1b).

**Table 2:** AIC_c_ rankings and weights of models describing the effects of taxonomic class (fish, amphibian, reptile, bird, mammal) and factorial aerobic scope (FAS) on the intra‐specific correlation between minimum metabolism and exercise‐induced maximum metabolic rate (VO_2_max), cold‐induced summit metabolic rate (Msum), and daily energy expenditure (DEE)

	Model^a^	*k*	λ	LogLik	AIC_c_	ΔAIC_c_	*w*_i_
VO_2_max (*n* = 115)	Std	**3**	**0·1**	**−35·81**	**77·85**	**0·00**	**0·70**
	Std + Class	7	0·0	−34·01	83·07	5·22	0·05
	Std + FAS	4	0·1	−35·86	80·08	2·23	0·23
	Std + Class + FAS	8	0·0	−34·22	85·80	7·95	0·01
Msum (*n* = 31)	Std	3	0·5	3·70	−0·51	5·44	0·05
	Std + Class	**4**	**0·6**	**7·75**	−**5·95**	**0·00**	**0·70**
	Std + FAS	4	0·5	5·54	−1·54	4·41	0·08
	Std + Class + FAS	5	0·0	7·82	−3·24	2·71	0·18
DEE (*n* = 30)	**Std**	**3**	**0·0**	**−6·69**	**20·30**	**0·00**	**0·50**
	Std + Class	5	0·0	−6·16	24·83	4·53	0·05
	Std + FAS	4	0·0	−5·49	20·59	0·29	0·43
	Std + Class + FAS	6	0·0	−5·65	26·96	6·66	0·02

Shown are the number of correlations in the analysis (*n*), the amount of phylogenetic signal (λ), the number of parameters (*k*), the log likelihood (LogLik) of the model, the difference in Akaike's information criterion (ΔAIC_c_) between each model and the top‐ranked model (in bold), and the Akaike weights (*w*_i_) of each model.

a Standard model = Intercept + minMR, where minMR is categorical standard metabolic rate (SMR) or basal metabolic rate (BMR) vs. resting metabolic rate (RMR).

### Inter‐specific correlations

Pearson's correlation coefficients for the inter‐specific correlation between minMR and upperMR ranged from −0·48 to 0·76 for VO_2_max, from −0·17 to 0·87 for Msum, and from 0·07 to 0·92 for DEE (Figs 3 and 4). The meta‐analyses performed separately for these variables suggest that effects sizes, and therefore the inter‐specific correlations, are consistently positive (Fig. 5). In fact, 95% confidence intervals were greater than zero for all estimates and taxonomic classes, with the notable exception of VO_2_max in reptiles for which a single estimate was available (*n* = 9 species, Pearson's *r *=* *0·04; Appendix S2). Comparison between models for VO_2_max suggests that differences between effects sizes can be partly attributed to the statistical analyses employed to estimate inter‐specific correlations (Table 3), with analyses that corrected for phylogeny providing higher estimates than those that did not (*z *=* *3·78, *P *=* *0·0002). In contrast, the model with the best fit for Msum provides some support for differences between birds and mammals (*z *=* *−1·89, *P *=* *0·059), whereas comparisons between models for DEE give similar weights for different models and therefore had very little discriminatory power (Table 3).

**Table 3:** AIC_c_ rankings and weights of models describing the effects of analysis (phylogenetic vs. non‐phylogenetic) and taxonomic class (fish, amphibian, reptile, bird, mammal) on the inter‐specific correlation between minimum and exercise‐induced maximum metabolic rate (VO_2_max), cold‐induced summit metabolic rate (Msum), and daily energy expenditure (DEE)

	Model	*k*	LogLik	AIC_c_	ΔAIC_c_	*w*_i_
VO_2_max (*n* = 13)	Int	1	−12·61	27·60	9·89	0·01
	**Int + Analysis**	**2**	−**6·25**	**17·71**	**0·00**	**0·98**
	Int + Class	5	−4·04	26·66	8·95	0·01
	Int + Analysis + Class	6	−2·08	30·16	12·45	0·00
Msum (*n* = 12)	Int	1	−10·95	24·29	0·63	0·34
	Int + Analysis	2	−10·46	26·27	2·61	0·13
	**Int + Class**	**2**	−**9·16**	**23·66**	**0·00**	**0·46**
	Int + Analysis + Class	3	−9·18	27·35	3·69	0·07
DEE (*n* = 14)	Int	1	−7·03	16·39	0·62	0·23
	**Int + Analysis**	**2**	−**5·34**	**15·77**	**0·00**	**0·31**
	Int + Class	2	−5·79	16·67	0·90	0·20
	Int + Analysis + Class	3	−3·81	16·03	0·26	0·27

Shown are the number of correlations in the analysis (*n*), the number of parameters (*k*), the log likelihood (LogLik) of the model, the difference in Akaike's information criterion (ΔAIC_c_) between each model and the top‐ranked model (in bold), and the Akaike weights (*w*_i_) of each model.

Int, intercept; Analysis, dummy variable comparing phylogenetically vs. non‐phylogenetically corrected analyses.

### Intra‐specific vs. inter‐specific correlations

The magnitude of the association between minMR and upperMR was significantly higher at the inter‐specific compared to intra‐specific level when adjusted effect sizes for the different taxonomic classes were compared (Fig. 5; paired *t*‐test, *t*_8_ = 4·81, *P *=* *0·001), with grand mean effect sizes and 95% confidence intervals back‐transformed into Pearson's *r* corresponding to 0·180 (intervals between 0·025 and 0·383) and 0·594 (0·346–0·771) for intra‐specific and inter‐specific analyses, respectively. Nonetheless, intra‐specific and inter‐specific effect sizes were not correlated (Pearson's *r*_7_ = −0·159, *P *=* *0·683).

## Discussion

Our meta‐analysis reveals some important generalities. First, the results support a general association between minMR and upperMR that is evident in most cases. Mean adjusted effect sizes were positive for all traits across all taxonomic classes, and 95% confidence intervals for pooled estimates were consistently higher than zero in all analyses, with the notable exception of intra‐specific correlations involving VO_2_max. Second, our analyses demonstrate that the magnitude of the association between minMR and upperMR is also generally consistent across taxa. And third, the association between minMR and upperMR at the inter‐specific level is consistently higher and appears to be unrelated to estimates at the intra‐specific level, suggesting that correlations at these two levels are affected by different factors and convey different types of information regarding the overall relationship between minMR and upperMR.

The positive correlation between minMR and upperMR is in line with physiological models that posit a mechanistic link between these traits (Packard 1968; Bennett & Ruben 1979; Drent & Daan 1980; Taigen 1983; Hayes & Garland 1995; Koteja 2000). However, part‐whole correlation may also give rise to this pattern (Chayes 1971). Here, the intra‐specific correlation between minMR and DEE decreases with increasing FAS. Hence, we find partial support for the association between minMR and upperMR being driven by part‐whole correlation but no tangible evidence for a physiological limit. This outcome may partly explain why intra‐specific effect sizes are significantly higher for DEE than for VO_2_max (see Results), since VO_2_max is typically much higher than either Msum (Chappell *et al*. 2004; McKechnie & Swanson 2010; Swanson *et al*. 2012) or DEE (Song & Wang 2002; Chappell *et al*. 2007) and therefore a greater multiple of minMR.

Other factors may also account for the overall low intra‐specific correlations observed for VO_2_max. First, motivation can be an issue in measurements of exercise‐induced maximal performance (Losos, Creer & Schulte 2002) and could have a major impact on estimates of VO_2_max, but not Msum or DEE. Second, studies of VO_2_max often included a small number of individuals (Fig. 2, Appendix S4). For instance, 39% of the correlations compiled for VO_2_max were obtained with *N* < 20, compared to 6% for Msum and 23% for DEE; when these estimates are removed, the mean pooled effect size increases (and the ±95% confidence intervals drop) from 0·108 ± 0·363 shown in Fig. 4 to 0·152 ± 0·194. Consequently, minMR and VO_2_max might exhibit a positive intra‐specific correlation more often than reported, simply because it may be more difficult to establish such an association for this particular trait. Third, maximum metabolic rates are typically measured during or after intense exercise in postprandial individuals, but there is evidence that digestion can increase oxygen consumption rates during exhaustive exercise in some species (Bennett & Hicks 2001; Fu *et al*. 2009) but not others (Alsop & Wood 1997; Fu *et al*. 2009; Jackson *et al*. 2015). Thus, measures at peak exercise alone may underestimate the total maximum aerobic capacity of some species and therefore explain why, relative to DEE, there was weaker evidence for a positive correlation between minMR and VO_2_max. Finally, there is some evidence that the direction and magnitude of the correlation between minMR and upperMR can change due to individual variation in plasticity in response to environmental factors such as temperature, hypoxia, and salinity (Careau, Gifford & Biro 2014; Norin, Malte & Clark 2016). However, the majority of studies thus far test for a correlation between minMR and VO_2_max in only a single environment, so at present we are unable to tease apart the relative effects of these extrinsic factors on observed intra‐specific correlations.

An alternative, but not mutually exclusive, explanation is that the strength of this association is malleable and evolves along the phylogeny, as recently described by Nespolo *et al*. (2017). Hence, the negligible or even negative correlation reported in some studies may be an accurate representation for those species and taxonomic groups. The notion that the association between minMR and upperMR evolves is supported by several lines of evidence. First, a clear phylogenetic signal was detected in our analyses of correlations between minMR and each of VO_2_max and Msum (Table 2). Second, the comparison between the intra‐specific effect sizes of Msum for birds vs. mammals provides a very compelling example of how physiological differences between lineages may emerge. Whereas small mammals employ brown adipose tissue and non‐shivering thermogenesis to thermoregulate in the cold (Nespolo *et al*. 2001), birds lack this specialized tissue and rely more heavily on shivering to produce heat (Swanson 2010). A higher positive correlation between minMR and Msum in birds could therefore reflect larger maintenance costs of muscles, since the contribution of brown fat to BMR is negligible (Cannon & Nedergaard 2011), and/or tighter directional selection on reduced body mass due to flight restrictions. Consequently, the evolution of brown fat in mammals, with its inherently low maintenance costs and no mechanical power output, may underlie the disruption of an otherwise general constraint imposed by the association between minMR and upperMR. Finally, there is some evidence that the direction and magnitude of the correlation between minMR and VO_2_max can differ among species according to their respective life styles and thermal ecology (Gomes *et al*. 2004). However, given that minMR and VO_2_max are plastic traits and their intra‐specific association can change as a function of the environment (see above), further study is needed to measure and compare the metabolic rates of different species acclimated to common garden conditions to better elucidate the degree to which these metabolic traits are coupled across different environments.

In the light of these results, the higher effect sizes observed in inter‐specific studies is not entirely surprising. The range of variation in minimum, sustained, and maximum metabolic rates is larger across species, and mass‐corrected metabolic rates can vary up to an order of magnitude among species (Weibel *et al*. 2004; Hillman, Hancock & Hedrick 2013; Killen *et al*. 2016) compared to the three to fourfold variation typically observed among individuals within a species (Kvist & Lindström 2001; Labocha *et al*. 2004; Steyermark *et al*. 2005; Careau, Gifford & Biro 2014). Whereas it is generally unclear to what extent genetic variation underlies observed phenotypic trends in intra‐specific analyses, inter‐specific comparisons involve by definition a higher degree of genetic differentiation that should partly account for the increased variation in metabolic rates observed across species. Consequently, plastic responses to environmental factors such as food and temperature (McKechnie, Chetty & Lovegrove 2007; Auer *et al*. 2015a), which will effectively add noise to metabolic estimates, are expected to have a higher impact on the phenotypic variation across individuals than across species. Indeed, there is evidence at the intra‐specific level that environmental and genetic effects can cancel one another out, leading to no phenotypic correlation despite a strong positive genetic one between minMR and VO_2_max (Sadowska *et al*. 2005). As such, the statistical power to detect a phenotypic correlation between these variables is expected to be higher in inter‐specific relative to intra‐specific analyses (Konarzewski, Książek & Łapo 2005). Our results confirm this prediction for all estimates across all taxonomic classes (this is apparent in Fig. 5 after noting that the *x*‐axis range differs between intra‐ and inter‐specific analyses).

In conclusion, our meta‐analyses suggest that a positive association between minMR and upperMR – estimated as VO_2_max, Msum, or DEE – is pervasive across vertebrate lineages. This is in line with the observation that despite enormous variation in metabolic rates, FAS in vertebrates generally falls within a very narrow range (Bennett & Ruben 1979; Hinds *et al*. 1993; Killen *et al*. 2016). Our results, in combination with the relatively low variation in FAS, provide compelling evidence that minMR and upperMR often evolve in tandem. However, more studies are needed to assess the genetic basis of their association since phenotypic correlations do not always mirror genetic ones (Dohm, Hayes & Garland 2001; Sadowska *et al*. 2005). In addition, the mechanistic causes underlying this observation remain a matter of debate, partly because some taxonomic groups remain very poorly studied (e.g., sustained metabolism and DEE in ectotherms have received little attention in the literature; see Table 1). Whereas research at subordinate levels may reveal the physiological basis of such an association (Hulbert & Else 1999, 2000), more studies of organismal aerobic performance may shed light on the evolutionary causes and ecological consequences of this general constraint.

## Authors’ contributions

S.K.A. conceived of the study with input on its design from S.S.K. and E.L.R.; S.K.A. and S.S.K. surveyed the literature and collected the data; S.K.A. and E.L.R. analysed the data; S.K.A. drafted the manuscript. All authors contributed to manuscript revisions and gave final approval for publication.

## Data accessibility

Data and associated references are provided in Appendices S2 and S3.

## Supporting information


**Lay Summary**

**Appendix S1.** Vertebrate phylogeny.

**Appendix S2.** Intra‐specific studies.

**Appendix S3.** Inter‐specific studies.

**Appendix S4.** Funnel plots of effect sizes.
