# Supplementary material for: Resting vs. active: a meta‐analysis of the intra‐ and inter‐specific associations between minimum, sustained, and maximum metabolic rates in vertebrates
Source: Funct Ecol. 2017 May 2;31(9):1728–38. doi: 10.1111/1365-2435.12879 (PMC5600087; doi:10.1111/1365-2435.12879)
Supplement: Supplementary file 1 — Lay Summary [file FEC-31-1728-s001.pdf]

Functional Ecology

BRITISH ECOLOGICAL SOCIETY

Links between minimum and maximum energy expenditure in animals

Sonya K. Auer, Shaun S. Killen and Enrico L. Rezende

Does an idling Ferrari consume more fuel than a Volkswagen Beetle? A similar question applies to living organisms. Whether at rest or active, animals are constrained to operate within the energetic bounds determined by their minimum and maximum metabolic rates, which can differ considerably among individuals and species. The floor and ceiling of metabolic capacity in an animal may be mechanistically linked to each other if the former reflects the idling cost of the machinery needed to support the latter. A Ferrari, for example, needs a larger engine to attain a higher top speed than a Beetle, but this engine may cost more to run even when the car is not moving. In animals, increased muscle mass and other biochemical features that support high levels of activity may similarly increase resting costs. Here we conduct the first comprehensive assessment of the relationship between minimum and maximum metabolic rates across a diversity of vertebrate taxa (fish, amphibians, reptiles, birds, and mammals), analysing 176 published estimates of their correlation across individuals within a species and 41 estimates of their correlation across species. We found a general positive association between minimum and maximum metabolic rate that is shared among all vertebrates, suggesting that Ferraris such as hummingbirds or cheetahs spend more fuel at rest than Beetles such as chickens or sloths. In other words, power can be costly. This finding has major implications for our understanding of how different lifestyles evolve: while it may be advantageous to be highly active to search for food or capture prey, there also seems to be a high price to pay while at rest. In fact, some researchers speculate that this cost-benefit relationship underlies the evolution of warm-blooded birds and mammals whose metabolism at rest is so high that they can produce enough heat to remain warm at cold temperatures. Our results suggest that minimum and maximum metabolic rates are functionally linked and evolve together, providing some support to this hypothesis.

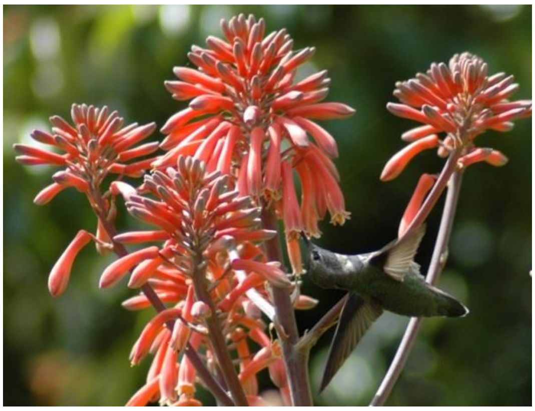

Hummingbird feeding. Photo provided by authors.
