# Supplementary material for: Resting vs. active: a meta‐analysis of the intra‐ and inter‐specific associations between minimum, sustained, and maximum metabolic rates in vertebrates
Source: Funct Ecol. 2017 May 2;31(9):1728–38. doi: 10.1111/1365-2435.12879 (PMC5600087; doi:10.1111/1365-2435.12879)
Supplement: Supplementary file 2 — Appendix S1. Vertebrate phylogeny. [file FEC-31-1728-s002.docx]

**Appendix S1**

Phylogenetic hypothesis employed in this study. Divergence times between the five major vertebrate clades included in our analyses were based on (Crottini *et al.* 2012), and the following sources were employed to build the backbone phylogeny for: (1) Fish: Near et al. (2012) and Betancur et al. (2013), relationships within Acanthomorpha and Cyprinidae followed Near et al. (2013) and Yang et al. (2015), respectively; (2) Amphibians: topology and divergence times followed Pyron and Wiens (2011) and Wiens (2007), respectively, and Wiens et al. (2010) for species within Hylidae; (3) Reptiles: topology from Pyron et al. (2013) and divergence times between Scincoidea, Anguimorpha and Serpentes based on Jones et al. (2013); (4) Mammals: the rodent phylogeny was obtained from Fabre et al. (2012) and the divergence time between Xenarthra and Rodentia from Arnason et al. (2008); and (5) Birds: topology and divergence time between major groups was based on Prum et al. (2015), relationships within Passeriformes on Jetz et al. (2012).


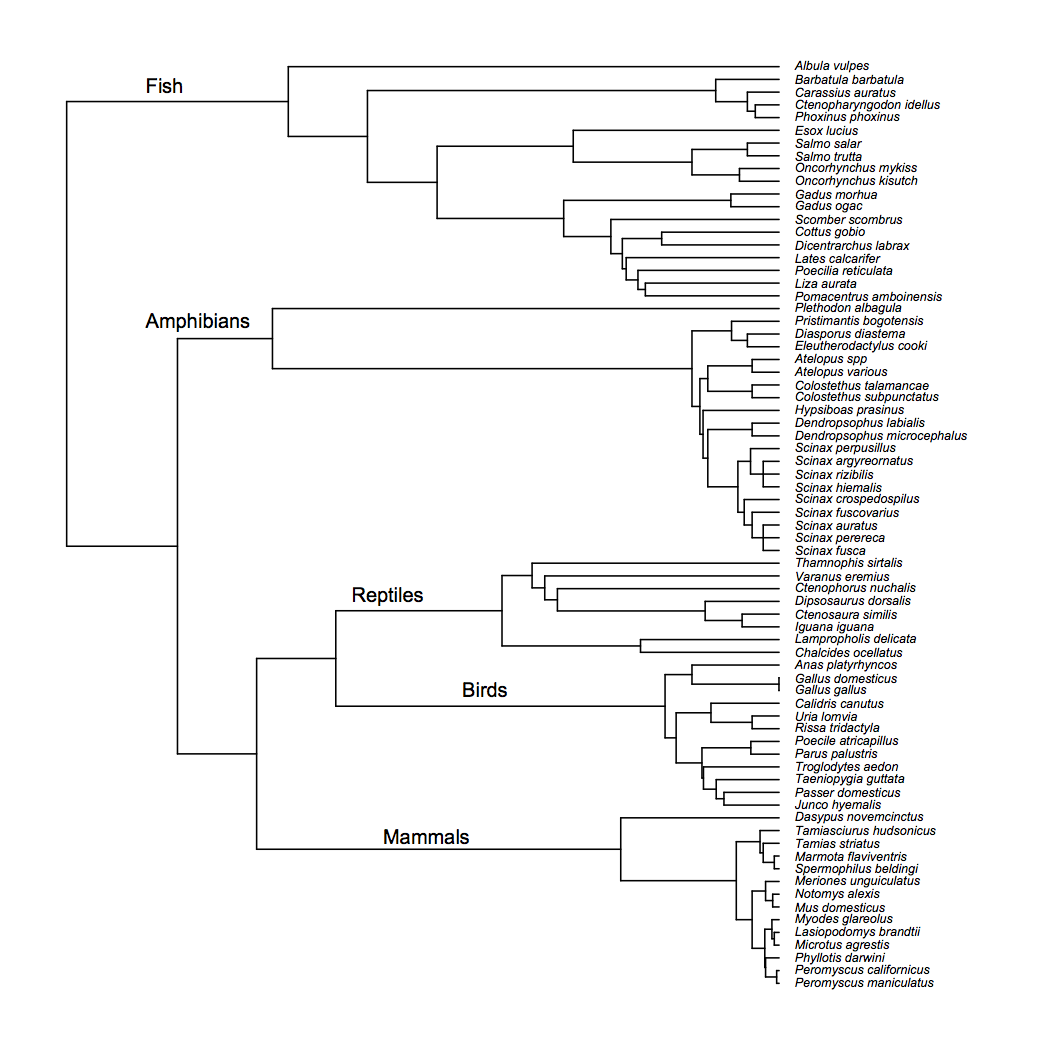


**References**

Arnason, U., Adegoke, J.A., Gullberg, A., Harley, E.H., Janke, A. & Kullberg, M. (2008) Mitogenomic relationships of placental mammals and molecular estimates of their divergences. *Gene,* **421,** 37-51.

Betancur, R., Broughton, R., Wiley, E., Carpenter, K., López, J., Li, C., Holcroft, N., Arcila, D., Sanciangco, M. & Cureton, J. (2013) The Tree of Life and a new classification of bony fishes. *PLOS Currents Tree of Life*.

Crottini, A., Madsen, O., Poux, C., Strauß, A., Vieites, D.R. & Vences, M. (2012) Vertebrate time-tree elucidates the biogeographic pattern of a major biotic change around the K–T boundary in Madagascar. *Proceedings of the National Academy of Sciences,* **109,** 5358-5363.

Fabre, P.-H., Hautier, L., Dimitrov, D. & Douzery, E.J. (2012) A glimpse on the pattern of rodent diversification: a phylogenetic approach. *BMC Evolutionary Biology,* **12,** 1.

Jetz, W., Thomas, G., Joy, J., Hartmann, K. & Mooers, A. (2012) The global diversity of birds in space and time. *Nature,* **491,** 444-448.

Jones, M.E., Anderson, C.L., Hipsley, C.A., Müller, J., Evans, S.E. & Schoch, R.R. (2013) Integration of molecules and new fossils supports a Triassic origin for Lepidosauria (lizards, snakes, and tuatara). *BMC Evolutionary Biology,* **13,** 208.

Near, T.J., Dornburg, A., Eytan, R.I., Keck, B.P., Smith, W.L., Kuhn, K.L., Moore, J.A., Price, S.A., Burbrink, F.T. & Friedman, M. (2013) Phylogeny and tempo of diversification in the superradiation of spiny-rayed fishes. *Proceedings of the National Academy of Sciences,* **110,** 12738-12743.

Near, T.J., Eytan, R.I., Dornburg, A., Kuhn, K.L., Moore, J.A., Davis, M.P., Wainwright, P.C., Friedman, M. & Smith, W.L. (2012) Resolution of ray-finned fish phylogeny and timing of diversification. *Proceedings of the National Academy of Sciences,* **109,** 13698-13703.

Prum, R.O., Berv, J.S., Dornburg, A., Field, D.J., Townsend, J.P., Lemmon, E.M. & Lemmon, A.R. (2015) A comprehensive phylogeny of birds (Aves) using targeted next-generation DNA sequencing. *Nature,* **526,** 569–573.

Pyron, R.A., Burbrink, F.T. & Wiens, J.J. (2013) A phylogeny and revised classification of Squamata, including 4161 species of lizards and snakes. *BMC Evolutionary Biology,* **13,** 1.

Pyron, R.A. & Wiens, J.J. (2011) A large-scale phylogeny of Amphibia including over 2800 species, and a revised classification of extant frogs, salamanders, and caecilians. *Molecular Phylogenetics and Evolution,* **61,** 543-583.

Wiens, J. (2007) Global patterns of species richness and diversification in amphibians. *American Naturalist,* **170,** S86-S106.

Wiens, J.J., Kuczynski, C.A., Hua, X. & Moen, D.S. (2010) An expanded phylogeny of treefrogs (Hylidae) based on nuclear and mitochondrial sequence data. *Molecular Phylogenetics and Evolution,* **55,** 871-882.

Yang, L., Sado, T., Hirt, M.V., Pasco-Viel, E., Arunachalam, M., Li, J., Wang, X., Freyhof, J., Saitoh, K. & Simons, A.M. (2015) Phylogeny and polyploidy: resolving the classification of cyprinine fishes (Teleostei: Cypriniformes). *Molecular Phylogenetics and Evolution,* **85,** 97-116.
