# Supplementary material for: Resting vs. active: a meta‐analysis of the intra‐ and inter‐specific associations between minimum, sustained, and maximum metabolic rates in vertebrates
Source: Funct Ecol. 2017 May 2;31(9):1728–38. doi: 10.1111/1365-2435.12879 (PMC5600087; doi:10.1111/1365-2435.12879)
Supplement: Supplementary file 3 — Appendix S2. Intra‐specific studies. [file FEC-31-1728-s003.docx]

**Appendix S2**

Intra-specific studies of the correlation (*r*) between minimum (minMR = SMR or BMR versus RMR) and different types of upperMR including exercise-induced maximum metabolic rate (VO_2_max), cold-induced summit metabolic rate (Msum), or daily energy expenditure (DEE). N = number of individuals in study. FAS = Factorial aerobic scope. NA = not available. Species whose names have changed since their publication date are denoted with a superscript with the previous Latin name detailed below.

| Species | minMR | upper MR | N | *r* | FAS | Reference |
| --- | --- | --- | --- | --- | --- | --- |
| **Fish** |  |  |  |  |  |  |
| *Albula vulpes* | SMR | VO_2_max | 7 | 0.896 | 2.37 | (Murchie *et al.* 2011) |
| *Barbatula barbatula* | SMR | VO_2_max | 16 | 0.182 | 4.59 | J. Nati, unpublished data |
| *Barbatula barbatula* | SMR | VO_2_max | 15 | 0.045 | 3.87 | J. Nati, unpublished data |
| *Barbatula barbatula* | SMR | VO_2_max | 14 | 0.299 | 3.07 | J. Nati, unpublished data |
| *Carassius auratus* | RMR | VO_2_max | 80 | 0.464 | 4.00 | (Huang *et al.* 2013) |
| *Phoxinus phoxinus* | RMR | VO_2_max | 43 | 0.264 | 5.56 | (Killen, Nati & Suski 2015) |
| *Phoxinus phoxinus* | SMR | VO_2_max | 43 | 0.416 | 11.09 | (Killen, Nati & Suski 2015) |
| *Phoxinus phoxinus* | RMR | VO_2_max | 26 | -0.210 | 4.08 | (Killen 2014) |
| *Phoxinus phoxinus* | SMR | VO_2_max | 26 | -0.460 | 6.66 | (Killen 2014) |
| *Ctenopharyngodon idellus* | SMR | VO_2_max | 81 | 0.259 | 1.58 | (Zhang *et al.* 2014) |
| *Esox lucius* | SMR | VO_2_max | 32 | 0.509 | 3.43 | (Simms 2000) |
| *Esox lucius* | SMR | VO_2_max | 32 | 0.077 | 3.77 | (Simms 2000) |
| *Esox lucius* | SMR | VO_2_max | 30 | 0.451 | 2.97 | (Simms 2000) |
| *Salmo salar* | SMR | VO_2_max | 452 | 0.023 | 3.83 | T. Van Leeuwen, unpublished data |
| *Salmo trutta* | SMR | VO_2_max | 120 | 0.260 | 6.37 | (Auer *et al.* 2015) |
| *Salmo trutta* | SMR | VO_2_max | 44 | 0.051 | 11.32 | S.K. Auer, unpublished data |
| *Salmo trutta* | SMR | VO_2_max | 44 | 0.065 | 7.58 | S.K. Auer, unpublished data |
| *Salmo trutta* | SMR | VO_2_max | 44 | 0.210 | 4.26 | S.K. Auer, unpublished data |
| *Salmo trutta* | SMR | VO_2_max | 44 | 0.000 | 3.44 | S.K. Auer, unpublished data |
| *Salmo trutta* | SMR | VO_2_max | 36 | 0.587 | 5.78 | (Norin & Malte 2011) |
| *Salmo trutta* | SMR | VO_2_max | 34 | 0.057 | 7.14 | (Norin & Malte 2011) |
| *Salmo trutta* | SMR | VO_2_max | 33 | 0.275 | 6.55 | (Norin & Malte 2011) |
| *Salmo trutta* | SMR | VO_2_max | 33 | 0.120 | 8.05 | (Norin & Malte 2011) |
| *Salmo trutta* | SMR | VO_2_max | 66 | 0.352 | 7.71 | (Norin & Malte 2012) |
| *Salmo trutta* | SMR | VO_2_max | 30 | 0.103 | 4.66 | T. Van Leeuwen, unpublished data |
| *Oncorhynchus mykiss* | SMR | VO_2_max | 25 | 0.369 | 3.21 | (Van Leeuwen, Rosenfeld & Richards 2011) |
| *Oncorhynchus kisutch* | SMR | VO_2_max | 26 | 0.305 | 3.45 | (Van Leeuwen, Rosenfeld & Richards 2011) |
| *Gadus morhua* | SMR | VO_2_max | 6 | 0.628 | 2.42 | (Bushnell *et al.* 1994) |
| *Gadus ogac* | SMR | VO_2_max | 6 | 0.794 | 2.52 | (Bushnell *et al.* 1994) |
| *Scomber scombrus* | SMR | VO_2_max | 18 | -0.107 | 6.22 | S.S. Killen, unpublished data |
| *Cottus gobio* | SMR | VO_2_max | 19 | -0.083 | 5.88 | J. Nati, unpublished data |
| *Cottus gobio* | SMR | VO_2_max | 13 | -0.455 | 4.16 | J. Nati, unpublished data |
| *Cottus gobio* | SMR | VO_2_max | 10 | -0.512 | 3.76 | J. Nati, unpublished data |
| *Dicentrarchus labrax* | SMR | VO_2_max | 39 | 0.651 | 3.63 | (Killen, Marras & McKenzie 2011) |
| *Lates calcarifer* | SMR | VO_2_max | 60 | -0.089 | 3.83 | (Norin, Malte & Clark 2016) |
| *Lates calcarifer* | SMR | VO_2_max | 60 | -0.167 | 3.68 | (Norin, Malte & Clark 2016) |
| *Lates calcarifer* | SMR | VO_2_max | 60 | 0.254 | 3.61 | (Norin, Malte & Clark 2016) |
| *Lates calcarifer* | SMR | VO_2_max | 60 | 0.262 | 4.02 | (Norin, Malte & Clark 2016) |
| *Lates calcarifer* | SMR | VO_2_max | 60 | 0.376 | 3.04 | (Norin, Malte & Clark 2016) |
| *Poecilia reticulata* | SMR | VO_2_max | 20 | 0.510 | 9.97 | S.S. Killen, unpublished data |
| *Liza aurata* | RMR | VO_2_max | 20 | 0.025 | 2.46 | (Killen *et al.* 2012) |
| *Liza aurata* | SMR | VO_2_max | 20 | 0.185 | 3.64 | (Killen *et al.* 2012) |
| *Liza aurata* | SMR | VO_2_max | 14 | 0.021 | 7.68 | (Killen *et al.* 2015) |
| *Liza aurata* | SMR | VO_2_max | 14 | 0.219 | 6.58 | (Killen *et al.* 2015) |
| *Liza aurata* | SMR | VO_2_max | 14 | 0.591 | 5.21 | (Killen *et al.* 2015) |
| *Pomacentrus amboinensis* | RMR | VO_2_max | 72 | 0.192 | 3.55 | (Killen *et al.* 2014) |
|  |  |  |  |  |  |  |
| **Amphibians** |  |  |  |  |  |  |
| *Plethodon albagula* | SMR | VO_2_max | 19 | -0.115 | 2.83 | (Careau, Gifford & Biro 2014) |
| *Plethodon albagula* | SMR | VO_2_max | 19 | 0.273 | 2.46 | (Careau, Gifford & Biro 2014) |
| *Plethodon albagula* | SMR | VO_2_max | 19 | 0.415 | 2.94 | (Careau, Gifford & Biro 2014) |
| *Plethodon albagula* | SMR | VO_2_max | 19 | 0.849 | 2.97 | (Careau, Gifford & Biro 2014) |
| *Pristimantis bogotensis^1^* | RMR | VO_2_max | 10 | -0.175 | 4.40 | (Gomes *et al.* 2004) |
| *Pristimantis bogotensis^1^* | RMR | VO_2_max | 10 | 0.291 | 2.65 | (Gomes *et al.* 2004) |
| *Diasporus diastema^2^* | RMR | VO_2_max | 9 | -0.552 | 2.81 | (Gomes *et al.* 2004) |
| *Eleutherodactylus cooki* | RMR | VO_2_max | 12 | 0.180 | 5.30 | (Rogowitz & Sánchez-Rivoleda 1999) |
| *Eleutherodactylus cooki* | RMR | VO_2_max | 12 | 0.350 | 4.10 | (Rogowitz & Sánchez-Rivoleda 1999) |
| *Eleutherodactylus cooki* | RMR | VO_2_max | 12 | 0.260 | 3.70 | (Rogowitz & Sánchez-Rivoleda 1999) |
| *Colostethus talamancae* | RMR | VO_2_max | 9 | 0.617 | 6.46 | (Gomes *et al.* 2004) |
| *Colostethus subpunctatus* | RMR | VO_2_max | 10 | -0.667 | 2.60 | (Gomes *et al.* 2004) |
| *Atelopus spp^4^* | RMR | VO_2_max | 10 | 0.521 | 3.02 | (Gomes *et al.* 2004) |
| *Atelopus various* | RMR | VO_2_max | 10 | -0.161 | 2.87 | (Gomes *et al.* 2004) |
| *Hypsiboas prasinus^5^* | RMR | VO_2_max | 9 | 0.021 | 5.61 | (Gomes *et al.* 2004) |
| *Dendropsophus labialis^6^* | RMR | VO_2_max | 10 | -0.207 | 3.98 | (Gomes *et al.* 2004) |
| *Dendropsophus labialis^6^* | RMR | VO_2_max | 10 | 0.411 | 3.55 | (Gomes *et al.* 2004) |
| *Dendropsophus microcephalus^7^* | RMR | VO_2_max | 8 | -0.261 | 4.72 | (Gomes *et al.* 2004) |
| *Scinax perpusillus^8^* | RMR | VO_2_max | 16 | -0.143 | 9.61 | (Gomes *et al.* 2004) |
| *Scinax argyreornatus* | RMR | VO_2_max | 11 | 0.436 | 13.62 | (Gomes *et al.* 2004) |
| *Scinax rizibilis* | RMR | VO_2_max | 11 | -0.295 | 8.76 | (Gomes *et al.* 2004) |
| *Scinax hiemalis* | RMR | VO_2_max | 12 | 0.274 | 6.99 | (Gomes *et al.* 2004) |
| *Scinax crospedospilus* | RMR | VO_2_max | 8 | 0.649 | 13.90 | (Gomes *et al.* 2004) |
| *Scinax fuscovarius* | RMR | VO_2_max | 16 | 0.309 | 12.02 | (Gomes *et al.* 2004) |
| *Scinax auratus* | RMR | VO_2_max | 6 | -0.837 | 9.27 | (Gomes *et al.* 2004) |
| *Scinax perereca* | RMR | VO_2_max | 16 | -0.834 | 6.47 | (Gomes *et al.* 2004) |
| *Scinax fusca^9^* | RMR | VO_2_max | 9 | -0.454 | 14.93 | (Gomes *et al.* 2004) |
|  |  |  |  |  |  |  |
|  |  |  |  |  |  |  |
| **Reptiles** |  |  |  |  |  |  |
| *Lampropholis delicata* | RMR | VO_2_max | 56 | 0.223 | 3.04 | (Merritt, Matthews & White 2013) |
| *Chalcides ocellatus* | RMR | VO_2_max | 28 | 0.077 | 5.73 | (Pough & Andrews 1984) |
| *Chalcides ocellatus* | SMR | VO_2_max | 28 | 0.089 | 8.15 | (Pough & Andrews 1984) |
| *Thamnophis sirtalis* | SMR | VO_2_max | 78 | -0.184 | 1.63 | (Peterson, Walton & Bennett 1998) |
| *Thamnophis sirtalis* | SMR | VO_2_max | 78 | -0.165 | 2.15 | (Peterson, Walton & Bennett 1998) |
| *Thamnophis sirtalis* | SMR | DEE | 78 | -0.099 | 4.65 | (Peterson, Walton & Bennett 1998) |
| *Thamnophis sirtalis* | SMR | DEE | 78 | 0.015 | 4.65 | (Peterson, Walton & Bennett 1998) |
| *Varanus eremius* | SMR | VO_2_max | 8 | -0.014 | 12.98 | (Thompson & Withers 1997) |
| *Amphibolurus nuchalis* | SMR | VO_2_max | 56 | 0.219 | 12.11 | (Garland Jr & Else 1987) |
| *Dipsosaurus dorsalis* | SMR | VO_2_max | 70 | 0.176 | 10.63 | (John-Alder 1984) |
| *Ctenosaura similis* | SMR | VO_2_max | 18 | 0.122 | 9.60 | (Garland 1984) |
| *Iguana iguana* | RMR | DEE | 6 | 0.460 | 3.10 | (Lichtenbelt *et al.* 1993) |
| *Iguana iguana* | SMR | DEE | 6 | 0.480 | 2.32 | (Lichtenbelt *et al.* 1993) |
|  |  |  |  |  |  |  |
| **Birds** |  |  |  |  |  |  |
| *Anas platyrhyncos* | RMR | Msum | 22 | 0.420 | 3.19 | (Moe, Stølevik & Bech 2005) |
| *Anas platyrhyncos* | RMR | Msum | 22 | 0.680 | 3.89 | (Moe, Stølevik & Bech 2005) |
| *Anas platyrhyncos* | RMR | Msum | 44 | 0.530 | 3.43 | (Moe, Stølevik & Bech 2005) |
| *Gallus domesticus* | RMR | Msum | 32 | 0.380 | 1.99 | (Konarzewski *et al.* 2000) |
| *Gallus domesticus* | RMR | Msum | 60 | 0.450 | 2.32 | (Konarzewski *et al.* 2000) |
| *Gallus domesticus* | RMR | Msum | 29 | 0.460 | 2.65 | (Konarzewski *et al.* 2000) |
| *Gallus gallus* | BMR | VO_2_max | 36 | -0.330 | 13.10 | (Hammond *et al.* 2000) |
| *Gallus gallus* | BMR | VO_2_max | 36 | -0.046 | 6.60 | (Hammond *et al.* 2000) |
| *Calidris canutus* | BMR | Msum | 24 | 0.138 | 7.39 | (Vézina *et al.* 2006) |
| *Calidris canutus* | BMR | Msum | 26 | -0.039 | 7.30 | (Vézina *et al.* 2006) |
| *Uria lomvia* | BMR | DEE | 52 | 0.173 | 3.61 | (Elliott *et al.* 2013) |
| *Rissa tridactyla* | BMR | DEE | 32 | 0.320 | 2.61 | (Elliott *et al.* 2013) |
| *Rissa tridactyla* | BMR | DEE | 27 | 0.240 | 3.94 | (Fyhn *et al.* 2001) |
|  |  |  |  |  |  |  |
| *Poecile atricapillus* | BMR | Msum | 200 | 0.367 | 5.63 | (Cortes *et al.* 2015) |
| *Poecile atricapillus* | BMR | Msum | 64 | 0.330 | 6.24 | (Lewden, Petit & Vézina 2012) |
| *Poecile atricapillus* | BMR | Msum | 64 | 0.360 | 6.24 | (Lewden, Petit & Vézina 2012) |
| *Poecile atricapillus* | BMR | Msum | 13 | 0.219 | 6.25 | (Swanson *et al.* 2012) |
| *Parus palustris* | BMR | DEE | 6 | 0.840 | 3.19 | (Nilsson 2002) |
| *Troglodytes aedon* | BMR | DEE | 27 | 0.450 | 2.20 | (Versteegh *et al.* 2008) |
| *Taeniopygia guttata* | RMR | DEE | 24 | 0.590 | 2.72 | (Vézina, Speakman & Williams 2006) |
| *Passer domesticus* | BMR | VO_2_max | 28 | -0.150 | 10.80 | (Careau *et al.* 2014) |
| *Passer domesticus* | BMR | VO_2_max | 60 | 0.037 | 10.80 | (Careau *et al.* 2014) |
| *Passer domesticus* | BMR | VO_2_max | 32 | 0.204 | 10.80 | (Careau *et al.* 2014) |
| *Passer domesticus* | BMR | VO_2_max | 36 | 0.230 | 9.56 | (Chappell, Bech & Buttemer 1999) |
| *Passer domesticus* | BMR | VO_2_max | 30 | 0.440 | 8.33 | (Chappell, Bech & Buttemer 1999) |
| *Passer domesticus* | BMR | Msum | 39 | 0.245 | 7.35 | (Swanson *et al.* 2012) |
| *Passer domesticus* | BMR | VO_2_max | 16 | 0.138 | 11.60 | (Zhang *et al.* 2015) |
| *Passer domesticus* | BMR | Msum | 18 | 0.134 | 7.53 | (Zhang *et al.* 2015) |
| *Junco hyemalis* | BMR | VO_2_max | 23 | 0.366 | 7.78 | (Swanson *et al.* 2012) |
| *Junco hyemalis* | BMR | Msum | 23 | 0.339 | 6.11 | (Swanson *et al.* 2012) |
|  |  |  |  |  |  |  |
| **Mammals** |  |  |  |  |  |  |
| *Dasypus novemcinctus* | BMR | Msum | 40 | 0.450 | 7.18 | (Boily 2002) |
| *Tamiasciurus hudsonicus* | RMR | DEE | 33 | 0.013 | 1.65 | (Larivee *et al.* 2010) |
| *Tamias striatus* | RMR | DEE | 17 | 0.410 | 3.22 | (Careau *et al.* 2013) |
| *Tamias striatus* | RMR | DEE | 16 | 0.280 | 3.22 | (Careau *et al.* 2013) |
| *Tamias striatus* | RMR | DEE | 17 | 0.070 | 3.22 | (Careau *et al.* 2013) |
| *Tamias striatus* | RMR | DEE | 22 | 0.480 | 3.22 | (Careau *et al.* 2013) |
| *Tamias striatus* | BMR | Msum | 20 | -0.530 | 8.10 | (Careau, Garant & Humphries 2012) |
| *Tamias striatus* | BMR | Msum | 20 | -0.160 | 8.10 | (Careau, Garant & Humphries 2012) |
| *Marmota flaviventris* | RMR | DEE | 15 | -0.129 | 6.90 | (Salsbury & Armitage 1994) |
| *Spermophilus beldingi* | BMR | VO_2_max | 95 | 0.310 | 6.32 | (Chappell, Bachman & Odell 1995) |
| *Spermophilus beldingi* | BMR | Msum | 95 | 0.005 | 6.53 | (Chappell, Bachman & Odell 1995) |
| *Meriones unguiculatus* | RMR | VO_2_max | 40 | -0.170 | 12.08 | (Chappell *et al.* 2007) |
| *Meriones unguiculatus* | RMR | DEE | 40 | 0.565 | 1.85 | (Chappell *et al.* 2007) |
| *Meriones unguiculatus* | BMR | VO_2_max | 27 | -0.101 | 10.56 | (Chappell *et al.* 2007) |
| *Meriones unguiculatus* | BMR | DEE | 27 | -0.099 | 1.62 | (Chappell *et al.* 2007) |
| *Meriones unguiculatus* | BMR | Msum | 20 | 0.062 | 5.59 | (Song & Wang 2001) |
| *Notomys alexis* | BMR | VO_2_max | 11 | -0.001 | 6.42 | (White, Matthews & Seymour 2006) |
| *Myodes glareolus^10^* | BMR | VO_2_max | 913 | 0.050 | 5.16 | (Sadowska *et al.* 2005) |
| *Myodes glareolus^10^* | BMR | VO_2_max | 706 | 0.020 | 5.16 | (Sadowska *et al.* 2005) |
| *Myodes glareolus^10^* | BMR | VO_2_max | 1017 | 0.050 | 5.16 | (Sadowska *et al.* 2005) |
| *Myodes glareolus^10^* | BMR | Msum | 868 | -0.050 | 5.44 | (Sadowska *et al.* 2005) |
| *Myodes glareolus^10^* | BMR | Msum | 661 | -0.100 | 5.45 | (Sadowska *et al.* 2005) |
| *Myodes glareolus^10^* | BMR | Msum | 950 | -0.060 | 5.45 | (Sadowska *et al.* 2005) |
| *Myodes glareolus* | BMR | VO_2_max | 332 | 0.090 | 4.12 | (Boratyński & Koteja 2009) |
| *Mus domesticus* | RMR | VO_2_max | 24 | 0.007 | 5.99 | (Rezende *et al.* 2005) |
| *Mus domesticus* | RMR | VO_2_max | 47 | 0.063 | 6.27 | (Rezende *et al.* 2005) |
| *Mus domesticus* | RMR | VO_2_max | 23 | 0.156 | 6.56 | (Rezende *et al.* 2005) |
| *Mus domesticus* | RMR | Msum | 24 | -0.144 | 7.88 | (Rezende *et al.* 2005) |
| *Mus domesticus* | RMR | Msum | 47 | 0.003 | 8.27 | (Rezende *et al.* 2005) |
| *Mus domesticus* | RMR | Msum | 23 | 0.023 | 8.66 | (Rezende *et al.* 2005) |
| *Mus domesticus* | RMR | DEE | 47 | 0.646 | 2.32 | (Rezende *et al.* 2009) |
| *Mus domesticus* | RMR | DEE | 49 | 0.710 | 2.66 | (Rezende *et al.* 2009) |
| *Mus domesticus* | BMR | VO_2_max | 340 | 0.040 | 6.56 | (Dohm, Hayes & Garland 2001) |
| *Mus domesticus* | BMR | VO_2_max | 60 | -0.069 | 6.44 | (Hayes, Garland Jr & Dohm 1992) |
| *Mus domesticus* | BMR | Msum | 42 | -0.110 | 5.78 | (Konarzewski, Sadowski & Jozwik 1997) |
| *Mus domesticus* | BMR | VO_2_max | 1334 | 0.130 | 7.82 | (Wone *et al.* 2009) |
| *Lasiopodomys brandti^11^* | BMR | DEE | 40 | 0.448 | 1.51 | (Song & Wang 2002) |
| *Lasiopodomys brandti^11^* | BMR | Msum | 40 | 0.191 | 4.72 | (Song & Wang 2002) |
| *Lasiopodomys brandti^11^* | RMR | DEE | 75 | 0.260 | 3.45 | (Speakman *et al.* 2003) |
| *Lasiopodomys brandti^11^* | RMR | DEE | 75 | 0.260 | 3.45 | (Speakman *et al.* 2003) |
| *Lasiopodomys brandti^11^* | RMR | DEE | 75 | 0.160 | 3.45 | (Speakman *et al.* 2003) |
| *Lasiopodomys brandti^11^* | BMR | DEE | 21 | 0.063 | 2.90 | (Meerlo *et al.* 1997) |
| *Phyllotis darwini* | BMR | Msum | 108 | 0.247 | 5.39 | (Nespolo, Arim & Bozinovic 2003) |
| *Phyllotis darwini* | BMR | Msum | 364 | -0.080 | 9.25 | (Nespolo *et al.* 2005) |
| *Peromyscus californicus* | RMR | DEE | 21 | 0.636 | 2.00 | (Dlugosz *et al.* 2012) |
| *Peromyscus californicus* | RMR | DEE | 25 | 0.609 | 1.88 | (Dlugosz *et al.* 2012) |
| *Peromyscus californicus* | BMR | VO_2_max | 21 | 0.674 | 7.61 | (Dlugosz *et al.* 2012) |
| *Peromyscus californicus* | BMR | VO_2_max | 25 | 0.359 | 6.29 | (Dlugosz *et al.* 2012) |
| *Peromyscus maniculatus* | BMR | VO_2_max | 81 | 0.222 | 6.85 | (Hammond, Chappell & Kristan 2002) |
| *Peromyscus maniculatus* | BMR | VO_2_max | 40 | 0.251 | 7.31 | (Hammond, Chappell & Kristan 2002) |
| *Peromyscus maniculatus* | BMR | VO_2_max | 12 | 0.277 | 5.86 | (Hammond, Chappell & Kristan 2002) |
| *Peromyscus maniculatus* | BMR | VO_2_max | 14 | -0.018 | 7.50 | (Hammond, Chappell & Kristan 2002) |
| *Peromyscus maniculatus* | BMR | VO_2_max | 15 | -0.048 | 6.75 | (Hammond, Chappell & Kristan 2002) |
| *Peromyscus maniculatus* | BMR | Msum | 50 | 0.318 | 10.76 | (Hayes 1989) |
| *Peromyscus maniculatus* | RMR | VO_2_max | 32 | 0.269 | 9.03 | (Chappell *et al.* 2004) |
| *Peromyscus maniculatus* | RMR | VO_2_max | 32 | 0.374 | 9.03 | (Chappell *et al.* 2004) |
| *Peromyscus maniculatus* | RMR | VO_2_max | 32 | -0.083 | 9.03 | (Chappell *et al.* 2004) |
| *Peromyscus maniculatus* | RMR | DEE | 32 | 0.632 | 1.89 | (Chappell *et al.* 2004) |
| *Peromyscus maniculatus* | RMR | DEE | 32 | 0.637 | 1.85 | (Chappell *et al.* 2004) |
| *Peromyscus maniculatus* | RMR | DEE | 32 | 0.869 | 2.47 | (Chappell *et al.* 2004) |

^1^ Eleutherodactylus bogotensis; ^2^ Eleutherodactylus diastema; ^3^ Bufo woodhousei fowleri; ^4^ Atelopus sp nov; ^5^ Hyla prasina;

^6^ Hyla labialis; ^7^ Hyla microcephala; ^8^ Scinax spp 3; ^9^ Scinax spp2; ^10^ Clethrionomys glareolus; ^11^ Microtus brandti;

**References**

Auer, S.K., Salin, K., Rudolf, A.M., Anderson, G.J. & Metcalfe, N.B. (2015) The optimal combination of standard metabolic rate and aerobic scope for somatic growth depends on food availability. *Functional Ecology,* **29,** 479-486.

Boily, P. (2002) Individual variation in metabolic traits of wild nine-banded armadillos (Dasypus novemcinctus), and the aerobic capacity model for the evolution of endothermy. *Journal of Experimental Biology,* **205,** 3207-3214.

Boratyński, Z. & Koteja, P. (2009) The association between body mass, metabolic rates and survival of bank voles. *Functional Ecology,* **23,** 330-339.

Bushnell, P., Steffensen, J., Schurmann, H. & Jones, D. (1994) Exercise metabolism in two species of cod in arctic waters. *Polar Biology,* **14,** 43-48.

Careau, V., Garant, D. & Humphries, M.M. (2012) Free-ranging eastern chipmunks (Tamias striatus) infected with bot fly (Cuterebra emasculator) larvae have higher resting but lower maximum metabolism. *Canadian Journal of Zoology-Revue Canadienne De Zoologie,* **90,** 413-421.

Careau, V., Gifford, M.E. & Biro, P.A. (2014) Individual (co) variation in thermal reaction norms of standard and maximal metabolic rates in wild‐caught slimy salamanders. *Functional Ecology,* **28,** 1175-1186.

Careau, V., Hoye, B.J., O'Dwyer, T.W. & Buttemer, W.A. (2014) Among-and within-individual correlations between basal and maximal metabolic rates in birds. *The Journal of Experimental Biology,* **217,** 3593-3596.

Careau, V., Réale, D., Garant, D., Pelletier, F., Speakman, J.R. & Humphries, M.M. (2013) Context-dependent correlation between resting metabolic rate and daily energy expenditure in wild chipmunks. *The Journal of Experimental Biology,* **216,** 418-426.

Chappell, M., Bachman, G.C. & Odell, J. (1995) Repeatability of maximal aerobic performance in Belding’s Ground Squirrels, *Spermophilus beldingi*. *Functional Ecology,* **9,** 498-504.

Chappell, M.A., Bech, C. & Buttemer, W.A. (1999) The relationship of central and peripheral organ masses to aerobic performance variation in house sparrows. *The Journal of Experimental Biology,* **202,** 2269-2279.

Chappell, M.A., Garland, T., Rezende, E.L. & Gomes, F.R. (2004) Voluntary running in deer mice: speed, distance, energy costs and temperature effects. *Journal of Experimental Biology,* **207,** 3839-3854.

Chappell, M.A., Garland, T., Robertson, G.F. & Saltzman, W. (2007) Relationships among running performance, aerobic physiology and organ mass in male Mongolian gerbils. *Journal of Experimental Biology,* **210,** 4179-4197.

Cortes, P.A., Petit, M., Lewden, A., Milbergue, M. & Vezina, F. (2015) Individual inconsistencies in basal and summit metabolic rate highlight flexibility of metabolic performance in a wintering passerine. *Journal of Experimental Zoology Part A: Ecological Genetics and Physiology,* **323,** 179-190.

Dlugosz, E.M., Harris, B.N., Saltzman, W. & Chappell, M.A. (2012) Glucocorticoids, aerobic physiology, and locomotor behavior in california mice*. *Physiological and Biochemical Zoology,* **85,** 671-683.

Dohm, M.R., Hayes, J.P. & Garland, T. (2001) The quantitative genetics of maximal and basal rates of oxygen consumption in mice. *Genetics,* **159,** 267-277.

Elliott, K.H., Welcker, J., Gaston, A.J., Hatch, S.A., Palace, V., Hare, J.F., Speakman, J.R. & Anderson, W.G. (2013) Thyroid hormones correlate with resting metabolic rate, not daily energy expenditure, in two charadriiform seabirds. *Biology open,* **2,** 580-586.

Fyhn, M., Gabrielsen, G.W., Nordøy, E.S., Moe, B., Langseth, I. & Bech, C. (2001) Individual variation in field metabolic rate of kittiwakes (*Rissa tridactyla*) during the chick‐rearing period. *Physiological and Biochemical Zoology,* **74,** 343-355.

Garland Jr, T. & Else, P. (1987) Seasonal, sexual, and individual variation in endurance and activity metabolism in lizards. *American Journal of Physiology,* **252,** R439-R449.

Garland, T. (1984) Physiological correlates of locomotory performance in a lizard: an allometric approach. *American Journal of Physiology-Regulatory, Integrative and Comparative Physiology,* **247,** R806-R815.

Gomes, F.R., Chauí‐Berlinck, J.G., Bicudo, J.E.P. & Navas, C.A. (2004) Intraspecific relationships between resting and activity metabolism in anuran amphibians: influence of ecology and behavior. *Physiological and Biochemical Zoology,* **77,** 197-208.

Hammond, K., Chappell, M. & Kristan, D. (2002) Developmental plasticity in aerobic performance in deer mice (Peromyscus maniculatus). *Comparative Biochemistry and Physiology Part A: Molecular & Integrative Physiology,* **133,** 213-224.

Hammond, K.A., Chappell, M.A., Cardullo, R.A., Lin, R.-s. & Johnsen, T.S. (2000) The mechanistic basis of aerobic performance variation in red junglefowl. *Journal of Experimental Biology,* **203,** 2053-2064.

Hayes, J., Garland Jr, T. & Dohm, M. (1992) Individual variation in metabolism and reproduction of Mus: are energetics and life history linked? *Functional Ecology,* **6,** 5-14.

Hayes, J.P. (1989) Altitudinal and seasonal effects on aerobic metabolism of deer mice. *Journal of Comparative Physiology B,* **159,** 453-459.

Huang, Q., Zhang, Y., Liu, S., Wang, W. & Luo, Y. (2013) Intraspecific scaling of the resting and maximum metabolic rates of the crucian carp (Carassius auratus). *PLoS ONE,* **8,** e82837.

John-Alder, H.B. (1984) Seasonal variations in activity, aerobic energetic capacities, and plasma thyroid hormones (T3 and T4) in an iguanid lizard. *Journal of Comparative Physiology B,* **154,** 409-419.

Killen, S.S. (2014) Growth trajectory influences temperature preference in fish through an effect on metabolic rate. *Journal of Animal Ecology,* **83,** 1513-1522.

Killen, S.S., Marras, S. & McKenzie, D.J. (2011) Fuel, fasting, fear: routine metabolic rate and food deprivation exert synergistic effects on risk‐taking in individual juvenile European sea bass. *Journal of Animal Ecology,* **80,** 1024-1033.

Killen, S.S., Marras, S., Steffensen, J.F. & McKenzie, D.J. (2012) Aerobic capacity influences the spatial position of individuals within fish schools. *Proceedings of the Royal Society B: Biological Sciences,* **279,** 357-364.

Killen, S.S., Mitchell, M.D., Rummer, J.L., Chivers, D.P., Ferrari, M.C., Meekan, M.G. & McCormick, M.I. (2014) Aerobic scope predicts dominance during early life in a tropical damselfish. *Functional Ecology,* **28,** 1367-1376.

Killen, S.S., Nati, J.J.H. & Suski, C.D. (2015) Vulnerability of individual fish to capture by trawling is influenced by capacity for anaerobic metabolism. *Proceedings of the Royal Society B-Biological Sciences,* **282,** 50603-50603.

Killen, S.S., Reid, D., Marras, S. & Domenici, P. (2015) The interplay between aerobic metabolism and antipredator performance: vigilance is related to recovery rate after exercise. *Frontiers in physiology,* **6,** 111.

Konarzewski, M., Gavin, A., McDevitt, R. & Wallis, I.R. (2000) Metabolic and organ mass responses to selection for high growth rates in the domestic chicken (Gallus domesticus). *Physiological and Biochemical Zoology,* **73,** 237-248.

Konarzewski, M., Sadowski, B. & Jozwik, I. (1997) Metabolic correlates of selection for swim stress-induced analgesia in laboratory mice. *American Journal of Physiology-Regulatory, Integrative and Comparative Physiology,* **273,** R337-R343.

Larivee, M.L., Boutin, S., Speakman, J.R., McAdam, A.G. & Humphries, M.M. (2010) Associations between over-winter survival and resting metabolic rate in juvenile North American red squirrels. *Functional Ecology,* **24,** 597-607.

Lewden, A., Petit, M. & Vézina, F. (2012) Dominant black-capped chickadees pay no maintenance energy costs for their wintering status and are not better at enduring cold than subordinate individuals. *Journal of Comparative Physiology B,* **182,** 381-392.

Lichtenbelt, W.D.V.M., Wesselingh, R.A., Vogel, J.T. & Albers, K.B. (1993) Energy budgets in free-living green iguanas in a seasonal environment. *Ecology,* **74,** 1157-1172.

Meerlo, P., Bolle, L., Visser, G.H., Masman, D. & Daan, S. (1997) Basal metabolic rate in relation to body composition and daily energy expenditure in the field vole, Microtus agrestis. *Physiological Zoology***,** 362-369.

Merritt, L., Matthews, P.G.D. & White, C.R. (2013) Performance correlates of resting metabolic rate in garden skinks Lampropholis delicata. *Journal of Comparative Physiology B-Biochemical Systemic and Environmental Physiology,* **183,** 663-673.

Moe, B., Stølevik, E. & Bech, C. (2005) Ducklings exhibit substantial energy‐saving mechanisms as a response to short‐term food shortage. *Physiological and Biochemical Zoology,* **78,** 90-104.

Murchie, K.J., Cooke, S.J., Danylchuk, A.J. & Suski, C.D. (2011) Estimates of field activity and metabolic rates of bonefish (Albula vulpes) in coastal marine habitats using acoustic tri-axial accelerometer transmitters and intermittent-flow respirometry. *Journal of Experimental Marine Biology and Ecology,* **396,** 147-155.

Nespolo, R.F., Arim, M. & Bozinovic, F. (2003) Body size as a latent variable in a structural equation model: thermal acclimation and energetics of the leaf-eared mouse. *Journal of Experimental Biology,* **206,** 2145-2157.

Nespolo, R.F., Bustamante, D.M., Bacigalupe, L.D. & Bozinovic, F. (2005) Quantitative genetics of bioenergetics and growth‐related traits in the wild mammal, Phyllotis darwini. *Evolution,* **59,** 1829-1837.

Nilsson, J.Å. (2002) Metabolic consequences of hard work. *Proceedings of the Royal Society of London. Series B: Biological Sciences,* **269,** 1735-1739.

Norin, T. & Malte, H. (2011) Repeatability of standard metabolic rate, active metabolic rate and aerobic scope in young brown trout during a period of moderate food availability. *Journal of Experimental Biology,* **214,** 1668-1675.

Norin, T. & Malte, H. (2012) Intraspecific variation in aerobic metabolic rate of fish: Relations with organ size and enzyme activity in Brown Trout. *Physiological and Biochemical Zoology,* **85,** 645-656.

Norin, T., Malte, H. & Clark, T.D. (2016) Differential plasticity of metabolic rate phenotypes in a tropical fish facing environmental change. *Functional Ecology,* **30,** 369-378.

Peterson, C.C., Walton, B.M. & Bennett, A.F. (1998) Intrapopulation variation in ecological energetics of the garter snake Thamnophis sirtalis, with analysis of the precision of doubly labeled water measurements. *Physiological and Biochemical Zoology,* **71,** 333-349.

Pough, F.H. & Andrews, R.M. (1984) Individual and sibling-group variation in metabolism of lizards: the aerobic capacity model for the origin of endothermy. *Comparative Biochemistry and Physiology Part A: Physiology,* **79,** 415-419.

Rezende, E.L., Chappell, M.A., Gomes, F.R., Malisch, J.L. & Garland, T. (2005) Maximal metabolic rates during voluntary exercise, forced exercise, and cold exposure in house mice selectively bred for high wheel-running. *Journal of Experimental Biology,* **208,** 2447-2458.

Rezende, E.L., Gomes, F.R., Chappell, M.A. & Garland Jr, T. (2009) Running behavior and its energy cost in mice selectively bred for high voluntary locomotor activity. *Physiological and Biochemical Zoology,* **82,** 662-679.

Rogowitz, G.L. & Sánchez-Rivoleda, J. (1999) Locomotor performance and aerobic capacity of the cave coqui, Eleutherodactylus cooki. *Copeia,* **1,** 40-48.

Sadowska, E.T., Labocha, M.K., Baliga, K., Stanisz, A., Wróblewska, A.K., Jagusiak, W. & Koteja, P. (2005) Genetic correlations between basal and maximum metabolic rates in a wild rodent: consequences for evolution of endothermy. *Evolution,* **59,** 672-681.

Salsbury, C.M. & Armitage, K.B. (1994) Resting and field metabolic rates of adult male yellow-bellied marmots, Marmota flaviventris. *Comparative Biochemistry and Physiology Part A: Physiology,* **108,** 579-588.

Simms, L.D. (2000) Intraspecific variation in the metabolism of juvenile Atlantic salmon salmo salar and northern pike esox lucius. Durham University.

Song, Z. & Wang, D. (2001) Relationships between metabolic rates and body composition in the Mongolian gerbils (Meriones unguiculatus). *Dong wu xue bao.[Acta zoologica Sinica],* **48,** 445-451.

Song, Z. & Wang, D. (2002) Relationship between metabolic rate and organ size in Brandt's voles (Microtus brandti). *Acta theriologica sinica,* **23,** 230-234.

Speakman, J., Ergon, T., Cavanagh, R., Reid, K., Scantlebury, D. & Lambin, X. (2003) Resting and daily energy expenditures of free-living field voles are positively correlated but reflect extrinsic rather than intrinsic effects. *Proceedings of the National Academy of Sciences,* **100,** 14057-14062.

Swanson, D.L., Thomas, N.E., Liknes, E.T. & Cooper, S.J. (2012) Intraspecific correlations of basal and maximal metabolic rates in birds and the aerobic capacity model for the evolution of endothermy. *PLoS ONE,* **7,** e34271.

Thompson, G.G. & Withers, P.C. (1997) Standard and maximal metabolic rates of goannas (Squamata: Varanidae). *Physiological Zoology,* **70,** 307-323.

Van Leeuwen, T.E., Rosenfeld, J.S. & Richards, J.G. (2011) Adaptive trade‐offs in juvenile salmonid metabolism associated with habitat partitioning between coho salmon and steelhead trout in coastal streams. *Journal of Animal Ecology,* **80,** 1012-1023.

Versteegh, M.A., Helm, B., Dingemanse, N.J. & Tieleman, B.I. (2008) Repeatability and individual correlates of basal metabolic rate and total evaporative water loss in birds: a case study in European stonechats. *Comparative Biochemistry and Physiology Part A: Molecular & Integrative Physiology,* **150,** 452-457.

Vézina, F., Jalvingh, K.M., Dekinga, A. & Piersma, T. (2006) Acclimation to different thermal conditions in a northerly wintering shorebird is driven by body mass-related changes in organ size. *The Journal of Experimental Biology,* **209,** 3141-3154.

Vézina, F., Speakman, J.R. & Williams, T.D. (2006) Individually variable energy management strategies in relation to energetic costs of egg production. *Ecology,* **87,** 2447-2458.

White, C.R., Matthews, P.G. & Seymour, R.S. (2006) Balancing the competing requirements of saltatorial and fossorial specialisation: burrowing costs in the spinifex hopping mouse, Notomys alexis. *Journal of Experimental Biology,* **209,** 2103-2113.

Wone, B., Sears, M.W., Labocha, M.K., Donovan, E.R. & Hayes, J.P. (2009) Genetic variances and covariances of aerobic metabolic rates in laboratory mice. *Proceedings of the Royal Society B: Biological Sciences,* **276,** 3695-3704.

Zhang, Y., Eyster, K., Liu, J.-S. & Swanson, D.L. (2015) Cross-training in birds: cold and exercise training produce similar changes in maximal metabolic output, muscle masses and myostatin expression in house sparrows (Passer domesticus). *Journal of Experimental Biology,* **218,** 2190-2200.

Zhang, Y., Huang, Q., Liu, S., He, D., Wei, G. & Luo, Y. (2014) Intraspecific mass scaling of metabolic rates in grass carp (Ctenopharyngodon idellus). *Journal of Comparative Physiology B,* **184,** 347-354.
