# Supplementary material for: Resting vs. active: a meta‐analysis of the intra‐ and inter‐specific associations between minimum, sustained, and maximum metabolic rates in vertebrates
Source: Funct Ecol. 2017 May 2;31(9):1728–38. doi: 10.1111/1365-2435.12879 (PMC5600087; doi:10.1111/1365-2435.12879)
Supplement: Supplementary file 4 — Appendix S3. Inter‐specific studies. [file FEC-31-1728-s004.docx]

**Appendix S3**

Inter-specific studies of the correlation (*r*) between minimum and exercise-induced maximum metabolic rate (VO_2_max), cold-induced summit metabolic rate (Msum), and daily energy expenditure (DEE) across different vertebrate taxonomic groups using analyses (PHY) that did (P) or did not (NP) correct for phylogenetic relationships among species. N = Number of species used to calculate the correlation. MR = metabolic rate.

| Taxon | PHY | MR | *N* | *r* | Reference |
| --- | --- | --- | --- | --- | --- |
| Fish | P | VO2max | 131 | 0.72 | (Killen *et al.* 2016) |
| Anurans | NP | VO2max | 17 | 0.63 | (Taigen 1983) |
| Anurans | NP | VO2max | 9 | 0.15 | (Gomes 2002) |
| Anurans | NP | VO2max | 8 | -0.48 | (Gomes 2002) |
| Anurans | P | VO2max | 15 | 0.68 | (Walton 1993) |
| Anurans | P | VO2max | 15 | 0.76 | (Walton 1993) |
| Lizards | NP | VO2max | 9 | 0.04 | (Thompson & Withers 1997) |
| Passerines | NP | DEE | 9 | 0.417 | (Koteja 1991) |
| Seabirds | NP | DEE | 12 | 0.511 | (Koteja 1991) |
| Birds | NP | DEE | 26 | 0.44 | (Daan, Masman & Groenewold 1990) |
| Birds | P | Msum | 10 | 0.86 | (Dutenhoffer & Swanson 1996) |
| Birds | P | DEE | 28 | 0.34 | (Ricklefs, Konarzewski & Daan 1996) |
| Birds | P | Msum | 24 | 0.87 | (Rezende *et al.* 2002) |
| Birds | P | VO2max | 45 | 0.41 | (Wiersma, Chappell & Williams 2007) |
| Birds | P | Msum | 19 | -0.17 | (Wiersma, Chappell & Williams 2007) |
| Birds | NP | DEE | 23 | 0.483 | (Koteja 1991) |
| Birds | NP | DEE | 8 | 0.75 | (Koteja 1991) |
| Shrews | P | Msum | 13 | 0.25 | (Sparti 1992) |
| Shrews | P | Msum | 12 | 0.29 | (Sparti 1992) |
| Rodents | NP | VO2max | 18 | 0.44 | (Hinds & Rice-Warner 1992) |
| Rodents | NP | VO2max | 17 | 0.64 | (Hinds & Rice-Warner 1992) |
| Rodents | NP | Msum | 9 | 0.70 | (Hinds & Rice-Warner 1992) |
| Rodents | NP | Msum | 29 | 0.459 | (Bozinovic 1992) |
| Rodents | NP | DEE | 14 | 0.653 | (Koteja 1991) |
| Rodents | P | Msum | 57 | 0.50 | (Rezende *et al.* 2004) |
| Rodentia | NP | DEE | 11 | 0.9 | (White & Seymour 2004) |
| Rodentia | NP | Msum | 41 | 0.4 | (White & Seymour 2004) |
| Rodentia | NP | VO2max | 16 | 0.3 | (White & Seymour 2004) |
| Dasyuromorphia | NP | Msum | 6 | 0.0 | (White & Seymour 2004) |
| Marsupials | NP | DEE | 9 | 0.066 | (Koteja 1991) |
| Diprotodontia | NP | DEE | 4 | 0.92 | (White & Seymour 2004) |
| Diprotodontia | NP | Msum | 5 | 0.84 | (White & Seymour 2004) |
| Carnivora | NP | VO2max | 4 | 0.3 | (White & Seymour 2004) |
| Chiroptera | NP | DEE | 5 | 0.7 | (White & Seymour 2004) |
| Eutheria | NP | DEE | 18 | 0.831 | (Koteja 1991) |
| Mammals | NP | VO2max | 18 | -0.14 | (Koteja 1987) |
| Mammals | NP | DEE | 30 | 0.70 | (White & Seymour 2004) |
| Mammals | NP | Msum | 56 | 0.40 | (White & Seymour 2004) |
| Mammals | NP | VO2max | 21 | 0.3 | (White & Seymour 2004) |
| Mammals | P | DEE | 33 | 0.746 | (Ricklefs, Konarzewski & Daan 1996) |
| Mammals | NP | DEE | 27 | 0.561 | (Koteja 1991) |
| Mammals | P | DEE | 60 | 0.364 | (Speakman 2000) |

**References**

Bozinovic, F. (1992) Scaling of basal and maximum metabolic rate in rodents and the aerobic capacity model for the evolution of endothermy. *Physiological Zoology,* **65,** 921-932.

Daan, S., Masman, D. & Groenewold, A. (1990) Avian basal metabolic rates: their association with body composition and energy expenditure in nature. *American Journal of Physiology-Regulatory, Integrative and Comparative Physiology,* **259,** R333-R340.

Dutenhoffer, M.S. & Swanson, D.L. (1996) Relationship of basal to summit metabolic rate in passerine birds and the aerobic capacity model for the evolution of endothermy. *Physiological Zoology,* **69,** 1232-1254.

Gomes, F.R. (2002) Estudo comparativo das inter-relações entre comportamento, ecologia térmica e fisiologia metabólica no gênero Scinax (Anura: Hylidae). Instituto de Biociências da Universidade de São Paulo. Departamento de Fisiologia.

Hinds, D.S. & Rice-Warner, C.N. (1992) Maximum metabolism and aerobic capacity in heteromyid and other rodents. *Physiological Zoology,* **65,** 188-214.

Killen, S.S., Glazier, D.S., Rezende, E.L., Clark, T.D., Atkinson, D., Willener, A.S.T. & Halsey, L.G. (2016) Ecological influences and morphological correlates of resting and maximal metabolic rates across teleost fish species. *American Naturalist,* **187,** 592–606.

Koteja, P. (1987) On the relation between basal and maximum metabolic rate in mammals. *Comparative Biochemistry and Physiology Part A: Physiology,* **87,** 205-208.

Koteja, P. (1991) On the relation between basal and field metabolic rates in birds and mammals. *Functional Ecology***,** 56-64.

Rezende, E.L., Bozinovic, F., Garland Jr, T. & Merilä, J. (2004) Climatic adaptation and the evolution of basal and maximum rates of metabolism in rodents. *Evolution,* **58,** 1361-1374.

Rezende, E.L., Swanson, D.L., Novoa, F.F. & Bozinovic, F. (2002) Passerines versus nonpasserines: so far, no statistical differences in the scaling of avian energetics. *Journal of Experimental Biology,* **205,** 101-107.

Ricklefs, R.E., Konarzewski, M. & Daan, S. (1996) The relationship between basal metabolic rate and daily energy expenditure in birds and mammals. *American Naturalist,* **147,** 1047-1071.

Sparti, A. (1992) Thermogenic capacity of shrews (Mammalia, Soricidae) and its relationship with basal rate of metabolism. *Physiological Zoology,* **65,** 77-96.

Speakman, J.R. (2000) The cost of living: Field metabolic rates of small mammals. *Advances in Ecological Research,* **30,** 177-297.

Taigen, T.L. (1983) Activity metabolism of anuran amphibians: implications for the origin of endothermy. *American Naturalist,* **121,** 94-109.

Thompson, G.G. & Withers, P.C. (1997) Standard and maximal metabolic rates of goannas (Squamata: Varanidae). *Physiological Zoology,* **70,** 307-323.

Walton, B.M. (1993) Physiology and phylogeny: the evolution of locomotor energetics in hylid frogs. *American Naturalist,* **141,** 26-50.

White, C.R. & Seymour, R.S. (2004) Does basal metabolic rate contain a useful signal? mammalian BMR allometry and correlations with a selection of physiological, ecological, and life‐history variables. *Physiological and Biochemical Zoology,* **77,** 929-941.

Wiersma, P., Chappell, M.A. & Williams, J.B. (2007) Cold-and exercise-induced peak metabolic rates in tropical birds. *Proceedings of the National Academy of Sciences,* **104,** 20866-20871.
