# Supplementary material for: Resting vs. active: a meta‐analysis of the intra‐ and inter‐specific associations between minimum, sustained, and maximum metabolic rates in vertebrates
Source: Funct Ecol. 2017 May 2;31(9):1728–38. doi: 10.1111/1365-2435.12879 (PMC5600087; doi:10.1111/1365-2435.12879)
Supplement: Supplementary file 5 — Appendix S4. Funnel plots of effect sizes. [file FEC-31-1728-s005.docx]

**Appendix S4**

Effects sizes of the intra- specific and inter-specific correlations between minimum metabolic rate and each of exercise-induced maximum metabolic rate (VO_2_max = black), cold-induced summit metabolic rate (Msum = grey), and daily energy expenditure (DEE = white) as a function of log_10_-transformed sample size.
